# Microwave-Assisted Green Synthesis and Characterization of Silver Nanoparticles Using *Melia azedarach* for the Management of *Fusarium* Wilt in Tomato

**DOI:** 10.3389/fmicb.2020.00238

**Published:** 2020-03-10

**Authors:** Hina Ashraf, Tehmina Anjum, Saira Riaz, Shahzad Naseem

**Affiliations:** ^1^Institute of Agricultural Sciences, University of the Punjab, Lahore, Pakistan; ^2^Center of Excellence in Solid State Physics, University of the Punjab, Lahore, Pakistan

**Keywords:** silver nanoparticles, *Melia azedarach*, antifungal, green synthesis, microwave assisted

## Abstract

These days, research in agriculture is focusing on the theme of sustainability along with protection of agriculture produce. Nanotechnology in the agriculture sector aims for the enhancement of agricultural produce and the reduction of pesticides through providing innovative agrochemical agents and their novel delivery mechanisms. The current investigation involved the green synthesis of silver nanoparticles (AgNPs) from the aqueous leaf extract of *Melia azedarach* by following a microwave-assisted method to control *Fusarium oxysporum*, the causal agent of tomato wilt. Biosynthesized *Melia* leaf extract (MLE)-AgNPs were characterized by UV-visible spectroscopy, Fourier-transform infrared (FTIR) spectroscopy, X-ray diffraction (XRD), energy dispersive X-ray (EDX) spectrometry, dynamic light scattering (DLS), scanning electron microscopy (SEM), transmission electron microscopy (TEM), and zeta potential analysis. The intensity of the peak at 434 nm in UV-vis spectra, attributed to the surface plasmon resonance of MLE-AgNPs, changes with reaction parameters. TEM exhibits spherical shaped nanoparticles with an average particle size range from 12 to 46 nm. Efficient inhibition of *F. oxysporum*, the causal agent of tomato wilt, was achieved after exposure to MLE-AgNPs both *in vivo* and *in vitro*. *In vitro* studies exhibited repressed fungal mycelial growth with 79–98% inhibition as compared to the control. Significant increases in growth parameters of tomato seedlings were observed after treatment with biosynthesized nanoparticles as compared to *F. oxysporum*-infected plants grown without them under greenhouse conditions. Furthermore, SEM imaging was done to reveal the prominent damage on the cell wall of hyphae and spores after MLE-AgNP treatment. Propidium iodide (PI) staining of mycelium indicated the extent of cell death, causing irretrievable damage and disintegration of cellular membranes by altering the membrane permeability. Also, 2′,7′-dichlorofluorescin diacetate (DCFH-DA) fluorescence specifies intracellular reactive oxygen species (ROS) production in *F. oxysporum* after treatment with MLE-AgNPs. The current investigation suggested that biosynthesized nanoparticles can revolutionize the field of plant pathology by introducing an environment-friendly approach for disease management and playing a potential part in agriculture industry. However, to date, little work has been done to integrate nanotechnology into phytopathology so, this area of research is in need of adoption and exploration for the management of plant diseases.

## Introduction

Presently, the term “green synthesis” or “phytonanotechnology” has been coined for nanoparticle (NP) synthesis which has many advantages including its biocompatibility, scalability, and applicability by utilizing water which acts as a reduction medium (Noruzi, 2015). It has been recommended that vitamins, proteins, organic acids, amino acids, and secondary metabolites act like capping and stabilizing agents that reduce metal salts of synthesized NPs by playing a key role (Duan et al., 2015). Green synthesis ascends as an incipient approach which has more advantages over physical and chemicals ways of synthesizing NPs. Biological processes implicate fungal, bacterial, and plant enzymes that involve convoluted procedures for sustaining cell cultures under aseptic conditions while significant production of NPs was achieved by employing plant extracts, encompassing simplicity and applicability with low energy consumption; however, chemically synthesized NPs comprise of toxic reagents that remain as residues along with particles and ultimately nurture toxicity problems within human system (Qayyum et al., 2017; Oves et al., 2018).

In the last few decades, extensive research has been done on the effects of NPs on ecosystems including organisms and plants. Being an eco-friendly and a multifactorial biogenic material, application of NPs is receiving attention due to their specific physiochemical properties, serves as an inexpensive approach for assembling of innovative functional materials used almost in every area of science and technology like medicine, engineering, environment, and agriculture (Austin et al., 2014; Majdalawieh et al., 2014; Siripattanakul-Ratpukdi and Furhacker, 2014; Prasad et al., 2016; Vishwakarma et al., 2017). Due to the interdynamic properties of NPs, there are high opportunities to explore the potential of NPs while the nano-elicitive behavior of these minute particles may rely on their nature and methods of synthesis (Jasim et al., 2017).

Silver (Ag)NPs arise as potential antimicrobial agents by demonstrating stout antiviral, bacterial, fungal, and inflammatory activities which highlighted their significance (Qayyum et al., 2017). To our best acquaintance, for the development of novel antimicrobial agents to manage agricultural diseases, manipulations in the synthesis of AgNPs such as structural properties and surface coating are some of the methods which are being employed for enhancing strong phytopathogenic activities (Jamshidi and Ghanati, 2016; Kumari et al., 2017). Some reports describe antifungal activity of AgNPs on conidial development and fungal hyphae as well as presenting strong inhibitory effects of biosynthesized AgNPs against certain phytopathogens in fields in greenhouse conditions (Mishra et al., 2014; Madbouly et al., 2017). In some earlier studies, nanomaterials have been employed to boost plant germination, improve soil fertility, and enhance deprivation of pesticide wastes (Ghrair et al., 2010; Khot et al., 2012; El-Temsah et al., 2014). Among the different types of metals, AgNPs have been used extensively against different fungal plant pathogens, and their suppressive effects on growth and development have been investigated. Elgorban et al. (2016) observed antifungal activity of AgNPs in cotton plants. Nano-silver has also been applied to suppress soilborne diseases. AgNPs from *Artemisia absinthium* extracts have shown high effect against agriculturally imperative pathogens from the genus *Phytophthora.* Its single applications of 10 and 100 μg/ml of nano-silver resulted in 78–95% plant survival, while 5% survival was observed for untreated control (Ali M. et al., 2015). Inhibitory effect of various formulations of nanoscale silver was studied against *Colletotrichum gloeosporioides* and *Sclerotium cepivorum* (Jung et al., 2010; Aguilar-Mendez et al., 2011). Mallaiah (2015) observed 75–55% disease reduction by AgNPs in commercially available ornamental flower of *Crossandra* spp. infected by *Fusarium* wilt in pot culture.

Formerly, an extensive research had been done on the utilization of different plants for the synthesis of AgNPs including aloe vera leaf extract (Medda et al., 2015), *Azadirachta indica* leaf extract (Ahmed et al., 2016), *Protium serratum* leaf extract (Mohanta et al., 2017), *Mangifera indica* inflorescence aqueous extract (Qayyum et al., 2017), *Phoenix dactylifera* root hair extract (Oves et al., 2018), and *Caesalpinia ferrea* seed extract (Soares et al., 2018). However, it is still required to explore commercially feasible, economically stable, and eco-friendly safe routes for synthesis of AgNPs by utilizing various plant materials (Chung et al., 2016).

Tomato (*Solanum lycopersicum* L.) is the most imperious vegetable harvest on earth that ranked second after potato (Jensen et al., 2010; FAOSTAT, 2014). Tomatoes are cultivated in different areas of Pakistan, with 11.05 tons per hectare (GOP, 2013). *F. oxysporum* f. sp. *lycopersici*, responsible for wilt, is an unavoidable infection of the grown tomatoes. Being a soilborne disease, it is only controlled by using resistant varieties and soil fumigants, but both have their own constraints. The impact of these pesticides on our ecosystem has already raised many questions on their utilization. Therefore, there is an essential need to adopt alternative approaches for the management of pathogens. An adequate approach that is being actively investigated involves NPs, such as metal oxides, which are used to control soilborne fungi (Shenashen et al., 2017).

The current study reports green synthesis of AgNPs by using the aqueous extract of *M. azedarach* and evaluates its potential application as an antifungal agent against *F. oxysporum* causing tomato wilt without intrusion of any supplementary physical and chemical steps. Silver has been chosen because of its antimicrobial activities either in NP or ionic form as well as lesser toxicity to mammalian cells (Ali K. et al., 2015). Moreover, the efficacy of *Melia* leaf extract (MLE)-AgNPs against *Fusarium* in tomato roots was evaluated under laboratory and greenhouse conditions by comparing it with a commercially available fungicide. The effects of MLE-AgNPs on tomato growth parameters and antioxidant enzymes were studied. Green synthesis of metal NPs by using plant material proficiently proved to be an eco-friendly and cost-effective approach.

## Materials and Methods

### Collection of Plant Material and Procurement of Pathogen Inoculum

Fresh leaves of *Melia azedarach* were collected from the tree planted in the vicinity of Punjab University, New Campus. Silver nitrate (AgNO_3_, 99.0%) and potato dextrose agar (PDA) was purchased from Sigma-Aldrich. Pure culture of *F. oxysporum* IAGS-1322 was procured from the First Culture Bank of Pakistan (FCBP). The strain was maintained, subcultured monthly, and preserved on PDA medium in glass culture tubes at 4°C. All the solutions were prepared with deionized water. All glassware was rinsed and sterilized before use to avoid contamination.

### Preparation of *Melia azedarach* Leaf Extract

**FIGURE 1:**
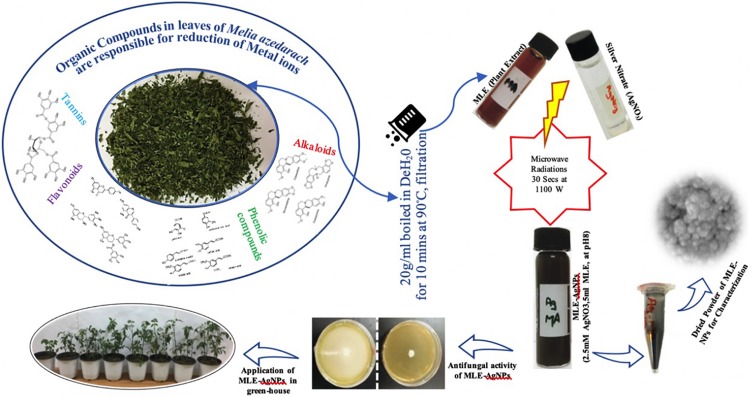
Graphical representation for green synthesis of silver nanoparticles (AgNPs) [*Melia* leaf extract (MLE)-AgNPs] from dried leaves of *Melia azedarach* and its applications.

The healthy and fresh leaves of *M. azedarach* were washed thoroughly with distilled water and then with deionized water to remove dust particles. Leaves were air-dried at room temperature for 3 days. Dried leaves (20 g) (Figure 1) were boiled in 100 ml deionized water at 90°C for 20 min in a temperature-controlled water bath. The leaves extract was cooled and filtered through Whatman filter paper. The extract was stored at 4°C and used within 2 weeks. This extract was used as a stabilizing and reducing agent.

### Synthesis of *Melia* Leaf Extract-Capped Silver Nanoparticles

The reaction conditions for the green synthesis of MLE-AgNPs were optimized. Typical reaction contained 5 ml of MLE, mixed with 45 ml of 2.5 mM AgNO_3_, pH 8.0 in a 100-ml flask. For rapid microwave synthesis, the synthesis product was subjected to a domestic microwave oven (Panasonic NN-CT651M) operating at the power of 1,100 W for a short pulse of 30 s. The mixture was then allowed to stand at room temperature for further use. The reaction conditions were optimized by performing the experiment for different conditions, wherein the reactions of MLE-AgNO_3_ were performed as the function of the concentrations of silver nitrate (0.5, 1, 1.5, 2, 2.5, 3, and 3.5 mM), amount of extract-MLE (1, 2, 3, 4, 5, 8, and 10 ml), pH (2, 4, 6, 8, and 10), and microwave irradiation (5, 10, 15, 30, 45, 60, and 75 s). All the reactions were performed at ambient temperature in dark conditions. A radical change in color from pale yellow to dark brown was observed. The biosynthesis of MLE-AgNPs was primarily detected by observing the color change from yellow to blackish brown.

### Characterization of *Melia* Leaf Extract-Capped Silver Nanoparticles

The biosynthesis of MLE-AgNPs was analyzed for surface plasmon resonance (SPR) by using UV-vis spectrophotometer (Denovix DS-C) in the wavelength range of 200–800 nm with the resolution of 1 nm. After the synthesis, NPs were separated by centrifugation of the solution at 6,000 rpm for 30 min. The supernatant was disposed to avoid unbound moieties. Pellet was dispersed in distilled water and purified by repeated centrifugation. The purified pellets were dried in a vacuum oven at 50°C for 12 h. Further, the dried powder was scrapped out and used for characterization. Fourier-transform infrared (FTIR) spectra for MLE-AgNPs were obtained in the range of 4,000–400 cm^–1^ with an FTIR (Thermo Scientific Nicolet 6700) by following the KBr pellet protocol. X-ray diffraction (XRD) analysis was carried out to reveal the crystallographic nature of biosynthesized MLE-AgNPs by using Philips PANalytical X’Pert Powder diffractometer with K_β_ filtered Cu (Kα) (1.5406 Å) radiations (operating voltage of 40 kV at 15 mA). The XRD spectra were recorded from 5° to 100° 2θ angles and step size of 0.2°. The morphology and size of the sample were investigated by using TESCAN Vega LMU-Variable pressure scanning electron microscope (SEM) and JEOL 2010F transmission electron microscope (TEM). Furthermore, the zeta potential and average size of MLE-AgNPs were determined at room temperature by dynamic light scattering (DLS, Malvern Zetasizer Nano ZS, UK). Elemental analysis was performed by using energy dispersive X-ray (EDX) spectrometry.

### Antifungal Activity of *Melia* Leaf Extract-Silver Nanoparticles

Nanoparticles were screened for antifungal activity by mycelium inhibition method. The medium was prepared, autoclaved (121°C for 15 min) and allowed to cool. *In vitro* antifungal assays were performed by using different concentrations of nanoparticles in Petri plates. After 24 h of incubation, agar plugs of uniform size (4 mm in diameter) from 3-day-old cultures of *F. oxysporum* were transferred to the center of each medium plate amended with different concentrations of NPs. Control plates were also prepared by using distilled water only. All the plates were incubated at 25°C for 7 days. All the assays were performed in triplicate. After the incubation period of the PDA plates with NPs, the growth of fungal mycelium will be measured by using the following equation:

$$Rateofinhibition\left(\%\right)=\frac{R-r}{R}\times 100$$

Where “R” represents the radial growth of fungal mycelium in control plates, and “r” is the radial growth of fungal mycelium in NP-treated plates (Kim et al., 2012).

### Analysis of Fungal Hyphae and Spore by Scanning Electron Microscopy After Treatment With *Melia* Leaf Extract-Silver Nanoparticles

The morphological changes in hyphae and spores of *F. oxysporum* without (control) and with (100 and 120 μg/ml) the treatment of MLE-AgNPs were investigated by using field emission scanning electron microscopy (FE-SEM). After 7 days of incubation period, mycelial disks were cut from the peripheral area of the fungal cultures, fixed with 2.5% glutaraldehyde at 4°C for 2 h, postfixed with 1% aqueous osmium tetroxide (OsO_4_) and washed with 0.1 M phosphate buffer (pH 7.8). Subsequently, the samples were discretely dehydrated in an ascending ethanol series from 30, 50, 70, 80, and 90% for 20 min in each aqueous solution. The final step was performed with 100% ethanol for 30 min twice, and then the dehydrated samples were dried in a vacuum oven. Finally, thin sections of the samples were placed on double adhesive carbon conductive tape and observed under an SEM (S-4800, Hitachi, Japan). For the study of morphological changes in spores, the fungal suspension was treated with MLE-AgNPs for 24 h at 28°C. After centrifugation, at 3,500 rpm for conidia, the condensed cells were fixed by using the same protocol.

### Viability Analysis and Reactive Oxygen Species Production Upon Treatment With *Melia* Leaf Extract-Silver Nanoparticles

Propidium iodide (PI, Sigma Aldrich) was used to investigate the cell viability after treatment with MLE-AgNPs. The viability was examined in mycelia of 7-day-old fungi (control and 100 and 120 μg/ml). The mycelium of each treatment was resuspended in phosphate buffered saline (PBS) (0.1 M, pH 7.8) and later treated with PI at a final concentration of 2.5 μg/ml for 15 min under dark conditions. Stained cells were washed thrice with PBS. To measure the accumulation of reactive oxygen species (ROS) in mycelia, 2′,7′-dichlorofluorescin diacetate (DCFH-DA, Sigma, Aldrich) assay was conducted. Fluorescence images were observed by using Zeiss LSM 7 Confocal laser scanning microscope (CLSM) integrated with the Axiovert 200M inverted microscope (Carl Zeiss, Germany). The images were captured by using an emission/excitation wavelength of 617/536 nm for PI and 552/488 nm for DCFH-DA tests, respectively.

### Efficacy of *Melia* Leaf Extract-Silver Nanoparticles Under Greenhouse Conditions

The *in vivo* effects of MLE-AgNPs on tomato seedlings were evaluated under greenhouse experiments. Sandy-clay soil was steam pasteurized and then compacted into 60-cm-diameter sterilized plastic pots, with 5 kg soil per pot. Conidial suspension of *F. oxysporum* was prepared in potato dextrose broth by using sterile distilled water by adjusting the final concentration at 106 spores/ml. One week before sowing, each pot was inoculated with 50 ml of fungal suspension and placed in the greenhouse at 30°C by keeping the soil moist until sowing.

Tomato seedlings cv. Rio Grande (25–30 days old) were treated by root dipping method in the respective concentrations (5, 10, 20, 40, 60, 80, 100,120, and 140 μg/ml) of MLE-AgNPs for 2 h (El-Mougy et al., 2013; El-Mohamedy et al., 2014). For the pathogenic control, the roots of the tomato seedlings were dipped in distilled water only, whereas the non-pathogenic control involved placing of seedlings in Nativo fungicide for 2 h. Each treatment was represented by three replicates. Later, the tomato seedlings were transplanted to soil inoculated with fungal suspension in the pots, with five plants per pot.

Two foliar sprays with 10 days of interval between each spray were applied on the tomato plants after 2 weeks of transplanting. After 45 days, the number of plants showing wilt symptoms, disease incidence, and growth parameters such as shoot and root length, biomass (fresh and dry weight), were recorded. The seedlings intact with roots were blotted to remove excess moisture, and the fresh biomass was determined. The same seedlings were dried in a hot air oven at 40°C for 72 h to obtain dry biomass (in grams). Disease incidence was calculated by using the following formula proposed by Vincent (1947).

$$\mathrm{Disease}\mathrm{Incidence}\left(\%\right)=\frac{\mathrm{Number}\,\mathrm{of}\,\mathrm{infected}\,\mathrm{plants}}{\mathrm{Total}\,\mathrm{number}\,\mathrm{of}\,\mathrm{plants}}\times 100$$

### Determination of Chlorophyll and Carotenoid Contents

Pocock et al. (2004) method with some modifications was used for the estimation of chlorophyll content in leaves. Fresh roots and shoots (0.1 g) of each treatment were crushed and then centrifuged at 10,000 rpm for 5 min with 1 ml of 100% acetone. Afterward, 0.95 ml of 80% acetone (2.5 mM sodium phosphate buffer pH 7.8) was added. One hundred percent acetone was used as a blank. By following the procedure of Porra (2002), the total content of chlorophyll a (Chl a) and chlorophyll b (Chl b) was determined by spectrometry by measuring absorbance at 664 and 647 nm and putting in Equations (1) and (2). Total chlorophyll is the sum of Chl “a” and “b.” By using Wellburn (1994) method, total carotenoids (TCs) were estimated by taking absorbance at 470 nm and the data of chlorophyll “a” and “b” in Equation (3). Results were calibrated in milligrams per gram fresh weight.

(1) Chl⁢a=12.25×Abs664-2.55×Abs647

(2) Chl⁢b=20.31×Abs647-4.91×Abs664

(3) TC=(1,000×Abs470-1.82×Chla-85.02×Chlb)/198

### Quantification of Total Phenolics and Stress Enzymes

Quantification of total phenolics and stress enzymes was performed after 45 days from sowing, and the harvested plants were separated into roots and shoots and used for estimating total phenolics, peroxidase (PO), polyphenoloxidase (PPO), and phenyl ammonia lyase (PAL) by the following methods.

### Quantification of Total Phenolics

Five milliliters of distilled water was taken in a clean test tube. A further 1 ml of methanolic extract and 250 μl of 50% Folin–Ciocalteu reagent was added inside this tube and kept for incubation in the dark for half an hour. After half an hour, 1 ml of 50% solution of sodium carbonate was added and incubated for a further 10 min inside the dark. After incubation, absorbance was measured using spectrophotometer at 725 nm. Standard curve was drawn using catechol. The quantity of total phenolics was given as micrograms catechol per milligram by comparing with the standard curve (Zieslin and Ben-Zaken, 1993).

### Quantification of Enzymes

One gram of plant material was crushed in a prechilled mortar containing 5 ml of ice- cold 100 mM phosphate buffer (pH 7). The homogenized material was centrifuged at 5,000 rpm at 4°C for 15 min. The above clear supernatant was collected and used for further quantification of enzymes.

### Quantification of Peroxidases

Fu and Huang (2001) method was used to determine the PO activity. Guaiacol was used as substrate. For this, 10 ml of 10 mM sodium phosphate buffer (pH 6.0) was added in 100 μl of hydrogen peroxide and mixed with 250 μl of guaiacol reagent. In the end, 3 ml of enzyme mixture was added and left for incubation at room temperature for 5 min. Absorbance was taken at 470 nm. PO activity was denoted as Δ470 nm/g fresh wt/min.

### Quantification of Polyphenol Oxidases

Polyphenoloxidase activity was quantified by method of Mayer et al. (1966). Catechol is used as a substrate to measure the enzyme activity. Reaction mixture was prepared by adding 1.5 ml of 10 mM sodium phosphate buffer (pH 6.0) to 150 μl of 0.1 M catechol solution and mixed. After this step, 200 μl of enzyme mixture was added to the tube and incubated it at room temperature for 1 h. Absorbance was taken at 495 nm. PPO activity was indicated as Δ495 nm min^–^1 mg−1 protein.

### Quantification of Phenylalanine Ammonia Lyase

Phenylalanine ammonia lyase (PAL) activity was measured by following the protocol of Burrell and Rees (1974). The reaction mixture contained 250 μl of 0.03 M L-phenylalanine and 200 μl of reaction mixture in a total of 2.5 ml of sodium borate buffer (pH 8.8). This reaction mixture was placed in a water bath at 37°C for 1 h.

After incubation period, 0.5 ml of 1.0 M trichloro acetic acid solution was added. Absorbance was noted at 290 nm, and enzyme activity was expressed as micrograms of *trans*-cinnamic acid per hour per milligram protein.

## Results

### Visual Observation and UV-Visible Spectroscopy of Microwave-Assisted *Melia* Leaf Extract-Silver Nanoparticles

**FIGURE 2:**
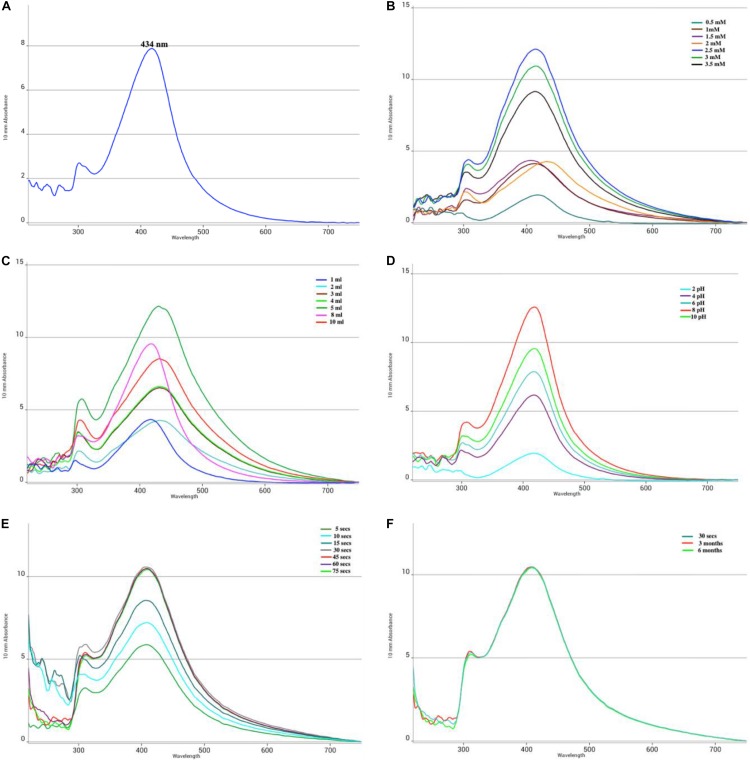
UV-visible absorption spectra of *Melia* leaf extract (MLE)-silver nanoparticles (AgNPs) synthesized by microwave-assisted protocol. UV-spectra under different optimization conditions during synthesis were documented as a function of **(A)** UV-visible absorption spectra of MLE-AgNPs; **(B)** silver nitrate (AgNO_3_) concentrations (0.5–3.5 mM); **(C)** amount of MLE (1–10 ml); **(D)** pH (2–10); **(E)** microwave irradiations (5–75 s); and **(F)** stability (30 s–6 months).

After exposure to microwave irradiation for a short pulse of 30 s, followed by 30 min incubation at room temperature, the color of the solution changes to dark blackish brown (Figure 1)indicating the generation of AgNPs. The peak at 434 nm in UV-vis spectrum is attributed to the SPR which is due to collective oscillations of the conduction of electrons of the MLE-AgNPs in the reaction solution which gradually surges with the exposure time (Figure 2A). However, in measurement range, no absorption peak was observed for control extract or silver ion solution. The solution does not contain much aggregated particles depicted from the symmetry of the plasmon band. Additionally, it is indicated from the results that green synthesis of MLE-AgNPs was significantly accelerated by approaching microwave irradiation.

The results in Figures 2B–F showed the optimization conditions customized for the microwave synthesis of MLE-AgNPs. The UV-vis spectra indicated that the absorbance values increased gradually as a function of MLE amount (1–10 ml), AgNO_3_ concentration (0.5–3.5 mM), pH (2–10), and microwave irradiation (5–75 s) time at ambient environment. Microwave irradiation affects the synthesis of MLE-AgNPs, aliquots of reaction solution were periodically subjected to UV-vis spectroscopy. Dark brown to black color was observed at higher salt concentration ranges from 2 to 3.5 mM while light brown color was observed from 0.5 to 1.5 mM. The SPR peaks became more distinct with increasing concentration of silver-nitrate, while the maximum peak intensity was obtained at 2.5 mM (Figure 2B). Surface plasmon absorbance increases with increasing MLE; however, maximum peak intensity was observed at 5 ml extract (Figure 2C) at pH 8 (Figure 2D). The absorbance intensity of the reaction solution exponentially increases with time. The Peak was observed after irradiation for 5 s, while its intensity increased with increasing reaction time; however, wavelength remained constant from 30 to 75 s as shown in Figure 2E. The microwave synthesis was completed in 30 s. There was no significant change detected in UV-visible spectrum and color of the biosynthesized MLE-AgNPs even after 3 and 6 months, exhibiting the well stability of nanoparticles in the solution (Figure 2F).

### Characterization of *Melia* Leaf Extract-Silver Nanoparticles

**FIGURE 3:**
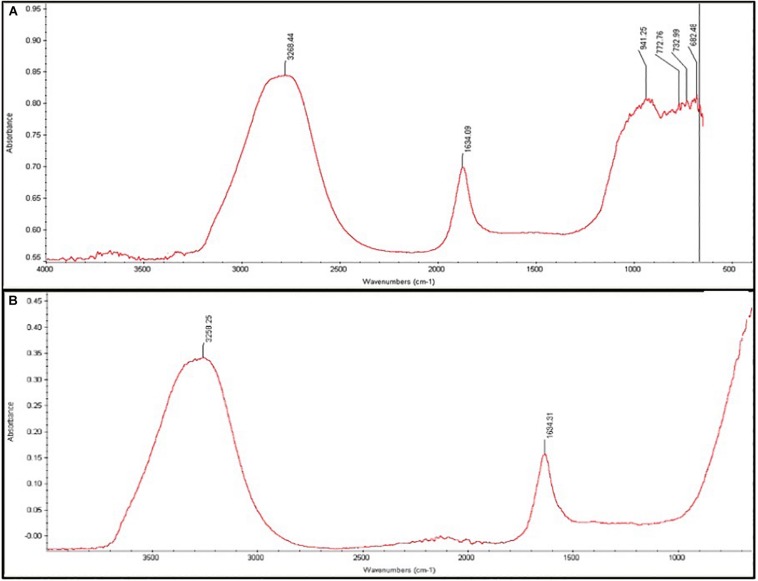
Fourier-transform infrared spectra of synthesized *Melia* leaf extract (MLE)-silver nanoparticles (AgNPs). **(A)** Spectra of MLE-extract alone. **(B)** Spectra of MLE-AgNPs.

Figures 3A,B show the FTIR spectra of *M. azedarach* extract and MLE- AgNPs. FTIR analyses have been done to evaluate the role of different phytochemicals of *M. azedarach* adsorbed at the surface which plays a key role in the synthesis and stability of AgNPs. A broad peak at about 3,258.25 represents vibrations of hydroxyl (–OH) group. While the absorption band at 1,634.31 cm^–1^ was attributed to variable stretching vibrations of alkene (C=C) with aromatic ring.

**FIGURE 4:**
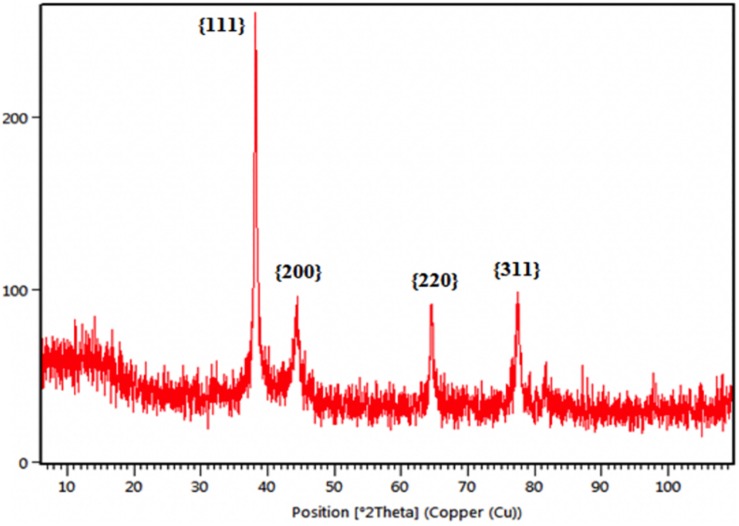
X-ray diffraction (XRD) pattern of *Melia* leaf extract-silver nanoparticles.

The crystalline nature of green synthesized MLE-AgNPs was determined by XRD analysis in the whole spectrum of 2θ values ranging from 10 to 100 as shown in Figure 4. The XRD pattern for the biosynthesized AgNPs revealed four intense peaks at 38.12°, 44.23°, 64.51°, and 77.69° that can be assigned to the plane of {111}, {200}, {220}, and {311}, respectively, and designates the face-centered-cubic (fcc) AgNPs and indicates the crystalline nature of the MLE-AgNPs (file JCPDS no. 04-0783). Debye-Scherrer’s equation was used to calculate the mean particle size of biosynthesized AgNPs based on full width at half maximum (FWHM) value for the {111} plane of reflection. The mean particle size of the MLE-AgNPs was consistent with transmission electron microscope (TEM) measurements.

**FIGURE 5:**
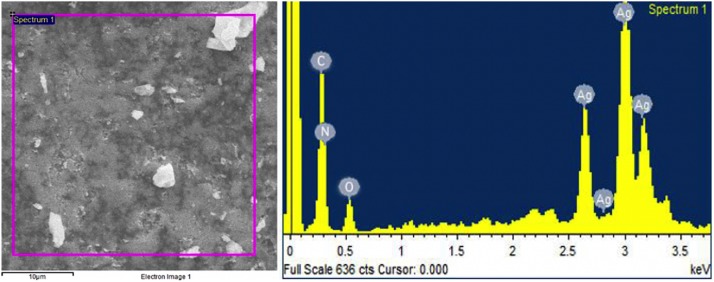
Represents the energy dispersive X-ray spectrometry spectrum of *Melia* leaf extract-silver nanoparticles.

Energy dispersive X-ray spectrometry (EDX) study confirmed the presence of elemental silver (23.62%), along with the signals of C, N, and O, as available in the reaction mixture of synthesized NPs. Figure 5 revealed the absorption peak at 3 keV region which showed that AgNPs were formed exclusively with crystalline nature.

**FIGURE 6:**
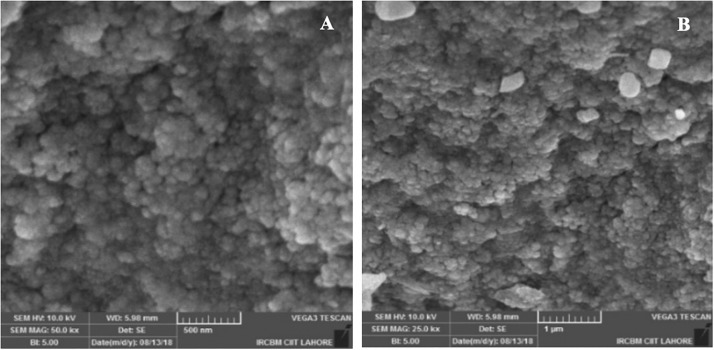
Scanning electron microscopic images of *Melia* leaf extract-silver nanoparticles at different magnifications **(A,B)**.

**FIGURE 7:**
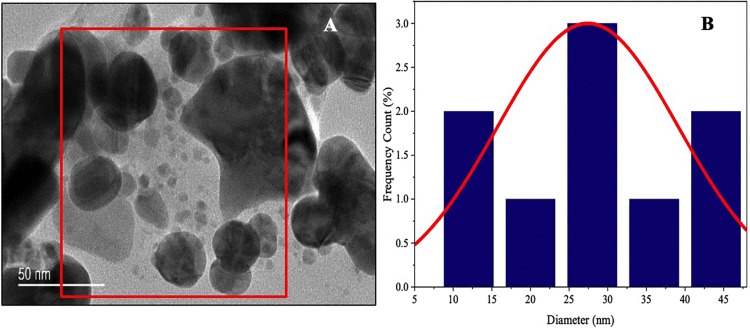
Morphological characterization of *Melia* leaf extract-silver nanoparticles (AgNPs) synthesized by microwave-assisted approach. **(A)** Transmission electron microscopic images of AgNPs (scale bar indicates 50 nm). **(B)** Histogram of size distribution of AgNPs.

To study the morphology and particle size, green synthesized AgNPs are further characterized by scanning electron microscope (SEM) analysis. The representative SEM images are shown in Figures 6A,B. The MLE-AgNPs obtained were mostly spherical in shape while other than spherical were also present. TEM images confirmed the synthesis of AgNPs by depicting variable and predominantly spherical and crystalline MLE-AgNPs with dark edges (Figure 7A). Figure 7B shows the histogram pattern of green synthesized AgNPs. The particle size distribution ranges from 12 to 46 nm with an average diameter of 28.04 nm.

**FIGURE 8:**
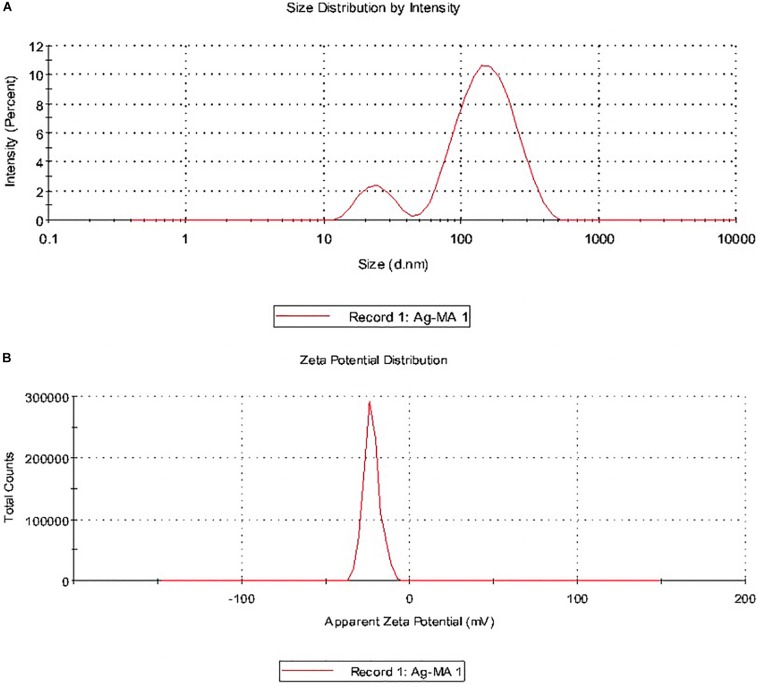
Size distribution intensity and zeta potential distribution of *Melia* leaf extract (MLE)-silver nanoparticles (AgNPs) **(A,B)** as revealed by dynamic light scattering.

Particle size and potential stability of AgNPs in colloidal suspension were determined by DLS and zeta potential, respectively. Figures 8A,B show the size distribution of MLE-AgNPs which was found to be in average of 98 nm, respectively. Moreover, the particles carried a charge of −22.3 mV. Therefore, biosynthesized MLE-AgNPs were stable at room temperature by showing a negative zeta potential.

### Antifungal Activity of *Melia* Leaf Extract-Silver Nanoparticles

**FIGURE 9:**
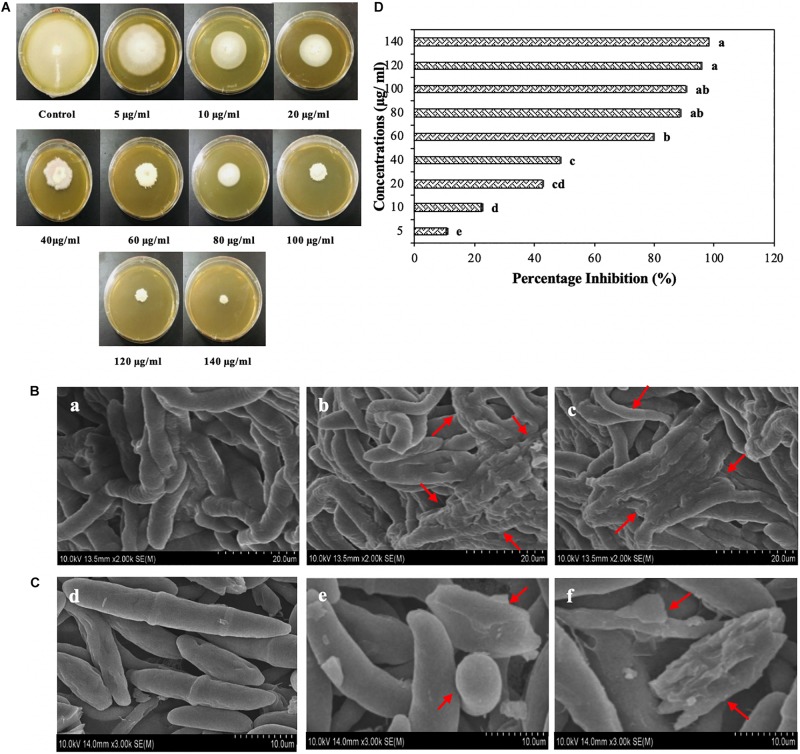
**(A)** Plates showing antifungal activity of *Melia* leaf extract (MLE)-silver nanoparticles (AgNPs) at different concentrations (5, 10, 20, 60, 80, 100, 120, and 140 μg/ml) after 7 days of incubation at 28°C. **(B)** Scanning electron microscope (SEM) images of *Fusarium oxysporum* hyphae in the presence of water **(a)**, 100 μg/ml **(b)** and 120 μg/ml of MLE-AgNPs **(c)**. **(C)** SEM images of conidia of *F. oxysporum* with sterile water **(d)** and MLE-AgNPs **(e,f)** for 24 h. **(D)** Effect of different concentrations (5, 10, 20, 40, 60, 80, 100, 120, and 140 μg/ml) of MLE-AgNPs after 7 days on *F. oxysporum* by calculating percentage inhibition (%). Vertical bars represent standard error between various replicates of the same treatments. Values with the same letter differ non-significantly (*P* ≥ 0.05) as created by ANOVA and Duncan’s new multiple range test.

The MLE-AgNPs expressively inhibited the growth of *F oxysporum* f. sp. *lycopersici* which clearly indicated that these NPs have the potential to be used as an active antifungal agent. Observations were recorded after 7 days for different concentrations (Figures 9A,D). It is evident from the results that higher concentrations (60–140 μg/ml) of AgNPs repressed fungal mycelial growth with 79–98% inhibition rate as compared to the control (*p* < 0.05). However, less than 50% inhibition was noted at lower concentrations, i.e., 5–40 μg/ml after the required incubation period in contrast to higher ones. The highest inhibition rate (98.2 ± 0.15) was observed at 140 μg/ml of MLE-AgNPs while the lowest rate (10.6 ± 0.379) was observed at 5 μg/ml.

### Hyphal and Spore Observation by Scanning Electron Microscope Induced by *Melia* Leaf Extract-Silver Nanoparticles

The effect of MLE-AgNPs on the mycelia and spores of *F. oxysporum* through SEM is shown in Figures 9B,C. Hyphae in the control treatment (treated with sterile water) indicates an intact and smooth exterior surface (Figure 9Ba), whereas after treatment with 100 μg/ml and 120 μg/ml of MLE-AgNPs, the mycelium shows a deformed shape with ruptured walls and hyphae became shrunk and stacked together (Figures 9Bb,c) which imbalances the mycelium integrity and ultimately inhibits fungus growth.

Experiments were conducted to observe alternation in spore morphology of *F. oxysporum* after treatment with MLE-AgNPs. In Figure 9Cd, untreated (control) macro-conidia depict slender, sickle to curved shape at ends, having an integral and enviable structure with transverse septa. However, after treatment with MLE-AgNPs (Figures 9Ce,f) for 24 h, most of the *F. oxysporum* macroconidia were creased, withered, and heaped together to form bumpy structures with NPs. Some large vesicles of non-germinated conidia are observed which impede spore germination. Furthermore, the normal cell shape was damaged, and macroconidia appeared to be highly disrupted, signifying the effect of NPs on the spores.

### Effect of *Melia* Leaf Extract-Silver Nanoparticles on the Viability and Reactive Oxygen Species Production in *Fusarium oxysporum*

**FIGURE 10:**
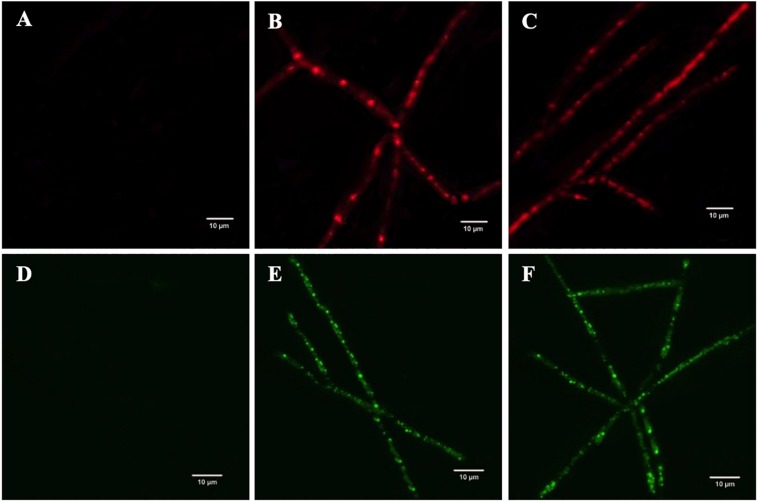
The effect of *Melia* leaf extract (MLE)-silver nanoparticles (AgNPs) on the production of intracellular reactive oxygen species (ROS) in *Fusarium oxysporum* mycelium, **(A)** control (sterile water), **(B)** 100 μg/ml, and **(C)** 120 μg/ml. Analyzing the propidium iodide influx on the membrane of *F. oxysporum* after treatment with MLE-AgNPs, **(D)** control, **(E)** 100 μg/ml, and **(F)** 120 μg/ml.

Fluorescence microscopy images acquired by CLSM were used to explicate the mechanism of fungal growth inhibition incited by MLE-AgNPs. Red color was accumulated by non-viable hyphae due to uptake of PI; however, control treatment did not show any detectable red-colored hyphae (Figure 10A), which reveals 100% viability of fungal mycelium, whereas in treated samples, the characteristic red-colored mycelium was detected at both concentrations, i.e., 100 and 120 μg/ml (Figures 10B,C). PI uptake experiment was compared with morphological changes indicated by SEM that NPs may alter the permeability of the cell membrane, causing disintegration, which ultimately induces cell death.

To study the intracellular ROS production in fungal mycelium, DCFH-DA fluorescence was used. Insignificant to very weak fluorescence was observed in the control sample (Figure 10D). Contrastingly, strong green color was detected in MLE-AgNP (100 μg/ml)-treated hyphae (Figure 10E). Furthermore, fluorescence intensity was amplified when *F. oxysporum* was treated at a higher concentration (120 μg/ml) of MLE-AgNPs (Figure 10F). This comparative analysis designates that green synthesized MLE-AgNPs increased ROS generation in the mycelium and damaged the cell membrane which results in fungal growth inhibition.

### Greenhouse Experiments for *in vivo* Efficacy Study of *Melia* Leaf Extract-Silver Nanoparticles

**FIGURE 11:**
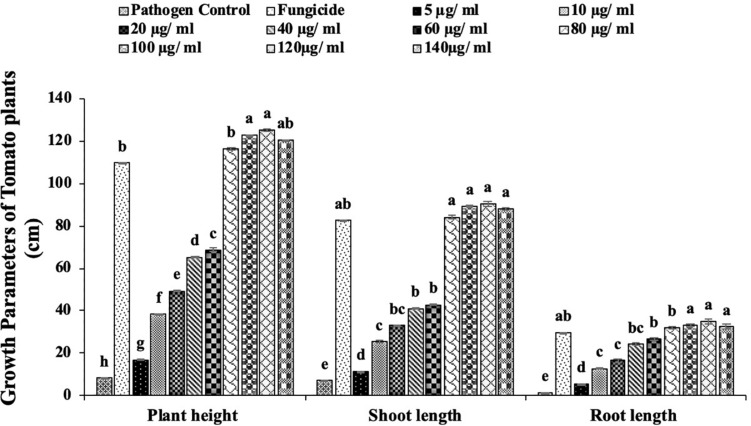
Effect of different concentrations of *Melia* leaf extract (MLE)-silver nanoparticles (AgNPs) on plant height and shoot and root length under greenhouse conditions against *Fusarium* wilt of tomato. Vertical bars represent standard error between various replicates of the same treatments. Values with the same letter differ non-significantly (*P* ≥ 0.05) as created by ANOVA and Duncan multiple range test.

**FIGURE 12:**
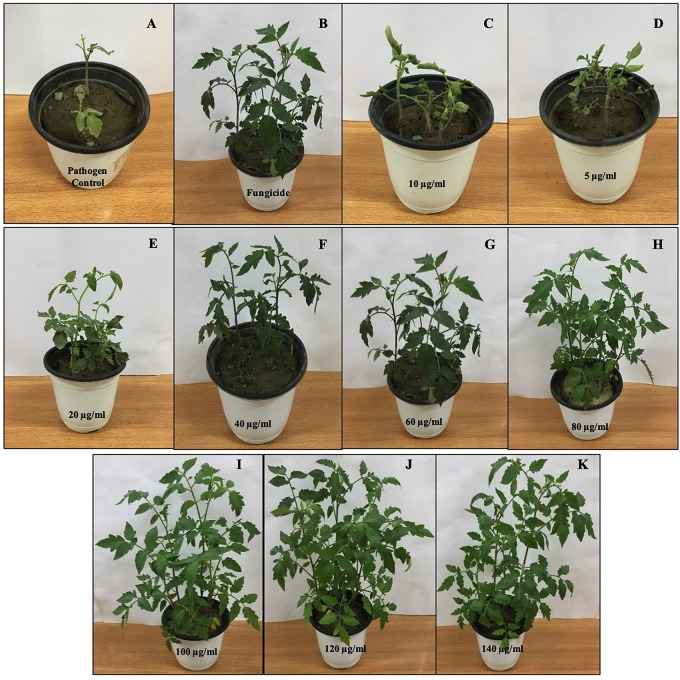
Effect of various concentrations of *Melia* leaf extract-silver nanoparticles (AgNPs) on disease development and vegetative growth of tomato plant after 45 days under greenhouse conditions infected with *Fusarium oxysporum*. **(A)** Pathogen control. **(B)** Fungicide. **(C–K)** Various concentrations (5, 10, 20, 40, 60, 80, 100, 120, and 140 μg/ml) of AgNPs.

The *in vivo* efficacy of MLE-AgNPs was evaluated in pots under greenhouse conditions against *F. oxysporum* causing tomato wilt (Figures 11, 12). The current study indicated significant improvement in growth parameters and disease reduction at different concentrations of AgNPs as compared to the pathogen and non-pathogen controls (Table 1).

**TABLE 1:** Evaluation of *Melia* leaf extract (MLE)-silver nanoparticles (AgNPs) under greenhouse conditions against *Fusarium* wilt of tomato.

Sr No.	NP concentrations (μg/ml)	Biomass (g)		Disease incidence (%)	Disease reduction (%)
		
		Fresh weight	Dry weight		
	Pathogen control	0.94 ± 0.08^d^	0.07 ± 0.07^c^	100 ± 0.05^a^	–
	Fungicide	16.2 ± 0.1^b^	5.68 ± 0.5^b^	11.2 ± 0.08^c^	89.8 ± 0.08^bc^
1	5	1.09 ± 0.06^cd^	0.97 ± 0.1^c^	68.6 ± 0.06^b^	31.4 ± 0.1^d^
2	10	3.03 ± 0.3^c^	1.17 ± 0.3^bc^	55.4 ± 0.1^bc^	44.6 ± 0.3^c^
3	20	7.95 ± 0.1^bc^	1.85 ± 0.09^b^	35.5 ± 0.09^c^	64.5 ± 0.07^bc^
4	40	9.59 ± 0.07^b^	2.09 ± 0.08^b^	28.2 ± 0.07^cd^	71.8 ± 0.06^b^
5	60	9.66 ± 0.4^b^	2.18 ± 0.4^ab^	25.6 ± 0.05^cd^	74.4 ± 0.09^b^
6	80	15.7 ± 0.2^a^	5.45 ± 0.5^a^	9.6 ± 0.02^d^	90.4 ± 0.08^ab^
7	100	16.8 ± 0.1^a^	5.87 ± 0.3^a^	0.0 ± 0.01^e^	100 ± 0.02^a^
8	120	18.7 ± 0.15^a^	6.43 ± 0.2^a^	0.0 ± 0.02^e^	100 ± 0.04^a^
9	140	16.3 ± 0.08^a^	5.67 ± 0.3^a^	0.0 ± 0.01^e^	100 ± 0.02^a^

*Mean values with the same letter differ non-significantly (*P* ≥ 0.05) as created by ANOVA and Duncan multiple range test.*

The growth parameters, i.e., plant height, root and shoot lengths, and fresh and dry plant weight, were taken in account for this study. It was noted that all concentrations of MLE-AgNPs showed effectiveness against disease activity by *F. oxysporum* and plays an active role in disease reduction and enhancement of growth parameters. An increase in concentrations from 20 to 140 μg/ml significantly reduces the disease, i.e., more than 50%, while at the highest concentrations from 100 to 140 μg/ml, no disease symptoms were observed. According to the current investigation, at the lowest concentration (5 μg/ml), the length of shoot, root, and plant height were 11.4, 5.5, and 16.9 cm as compared to the control that were estimated to be 6.9, 1.4, and 8.3 cm, respectively. Furthermore, fresh biomass and dry biomass of shoot and root were calculated to be 0.94 and 0.07 g in control, whereas 1.09 and 0.97g were noted for the lowest concentration. Increase in concentrations from 10 to 140 μg/ml was accompanied by significant disease reduction and improvement of plant health by affecting the growth parameters. However, in the case of pathogen control, 100% disease incidence was asserted. Observations regarding fungicide treatment (Nativo) were comparable to the higher concentrations, with 89.8% disease reduction. Growth parameters were also affected, so these findings suggested that NP treatments were more effective as compared to the commercially available fungicides. According to the current results, application of MLE-AgNPs at 120 μg/ml caused highest increment in growth parameters by causing complete inhibition of seedling wilt.

### Measurement of Chlorophyll and Carotenoid Contents

For plant growth, chlorophyll content has an important index. Table 2 shows that in comparison to the pathogen control, the total chlorophyll and carotenoid content significantly increased with MLE-AgNPs used in various concentrations. Our results showed that both chlorophyll “a” and “b” increased with higher concentrations. Total chlorophyll, i.e., 2.76 ± 0.18 and 2.81 ± 0.17, and carotenoid contents, i.e., 0.99 ± 0.18 and 1.11 ± 0.15, in leaves of tomato plants significantly increased at 100 and 120 μg/ml, respectively.

**TABLE 2:** Effect of various treatments of *Melia* leaf extract-silver nanoparticles on photosynthetic pigments of tomato plants.

Treatments μg/ml	Chlorophyll “a”	Chlorophyll “b”	Total chlorophyll	Total carotenoid
Pathogen control	0.57 ± 0.02^c^	0.186 ± 0.26^c^	0.76 ± 0.23^d^	0.06 ± 0.26^c^
Fungicide	1.56 ± 0.01^ab^	0.23 ± 0.67^bc^	1.79 ± 0.17^b^	0.49 ± 0.18^b^
5	0.52 ± 0.17^c^	0.17 ± 0.43^c^	1.08 ± 0.12^c^	0.03 ± 0.39^c^
10	0.89 ± 0.23^bc^	0.19 ± 0.18^c^	1.08 ± 0.18^c^	0.03 ± 0.27^c^
20	1.09 ± 0.28^b^	0.24 ± 0.17^bc^	1.33 ± 0.19^c^	0.36 ± 0.35^b^
40	1.65 ± 0.26^ab^	0.28 ± 0.19^bc^	1.93 ± 0.15^b^	0.42 ± 0.25^b^
60	1.87 ± 0.34^ab^	0.34 ± 0.37^b^	2.21 ± 0.16^ab^	0.74 ± 0.23^ab^
80	2.14 ± 0.32^a^	0.41 ± 0.19^ab^	2.55 ± 0.19^ab^	0.79 ± 0.19^ab^
100	2.25 ± 0.15^a^	0.51 ± 0.12^a^	2.76 ± 0.18^a^	0.99 ± 0.18^a^
120	2.27 ± 0.13^a^	0.53 ± 0.23^a^	2.81 ± 0.17^a^	1.11 ± 0.29^a^
140	2.21 ± 0.25^a^	0.48 ± 0.56^ab^	2.69 ± 0.18^a^	0.84 ± 0.27^ab^

*Mean values with the same letter differ non-significantly (*P* ≥ 0.05) as created by ANOVA and Duncan multiple range test.*

### Quantification of Total Phenolics and Stress Enzymes

**FIGURE 13:**
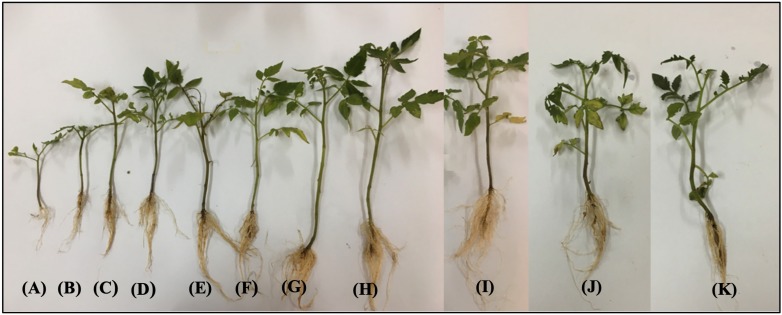
Effect of various concentrations of silver nanoparticles (AgNPs) on disease development and vegetative growth (root and shoot length) of tomato plant after 45 days under greenhouse conditions infected with *Fusarium oxysporum*. **(A)** Pathogenic control. **(B–J)** Different concentrations, i.e., 5, 10, 20, 40, 60, 80, 100, 120, and 140 mg/ml of *Melia* leaf extract (MLE)-AgNPs. **(K)** Fungicide.

**FIGURE 14:**
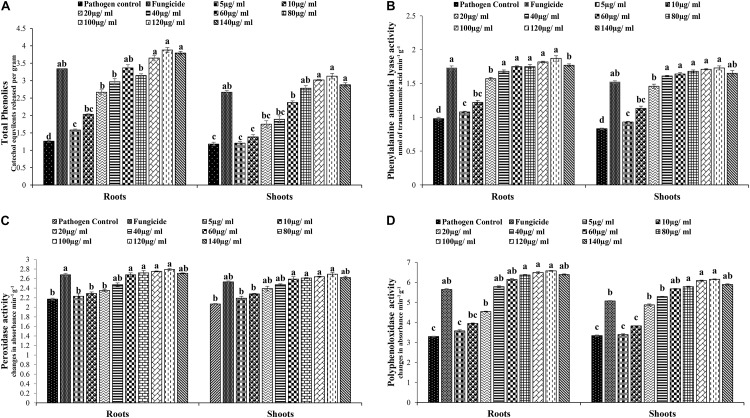
**(A)** Effect of various concentrations of *Melia* leaf extract (MLE)-silver nanoparticles (AgNPs) on total phenolics in the plants measured after 45 days. Vertical bars represent standard error between various replicates of the same treatments. Values with the same letter differ non-significantly (*P* ≥ 0.05) as created by ANOVA and Duncan multiple range test (DNMRT). **(B)** Effect of various concentrations of MLE-AgNPs on phenylalanine ammonia lyase (PAL) quantity in the plants measured after 45 days. Vertical bars represent standard error between various replicates of the same treatments. Values with the same letter differ non-significantly (*P* ≥ 0.05) as created by ANOVA and DNMRT. **(C)** Effect of various concentrations of MLE- AgNPs on peroxidase activity (PO) in the plants measured after 45 days. Vertical bars represent standard error between various replicates of the same treatments. Values with the same letter differ non-significantly (*P* ≥ 0.05) as created by ANOVA and DNMRT. **(D)** Effect of various concentrations of MLE-AgNPs on polyphenol oxidase activity (PPO) in the plants measured after 45 days. Vertical bars represent standard error between various replicates of the same treatments. Values with the same letter differ non-significantly (*P* ≥ 0.05) as created by ANOVA and DNMRT.

Application of different concentrations of AgNPs induced tomato plants for significantly (*P* > 0.05) higher production of phenolics, PAL, PO, and PPO as compared to the pathogen control (Figure 13). Phenolic compounds were quantified at different concentrations (Figure 14A). The highest phenolic quantities were observed at 120 μg/ml. Both in roots and shoots, the increase was 3.08- and 2.65-fold as compared to the pathogen control. The phenolic content after 45 days of sowing increased with increasing concentrations of MLE-AgNPs both in roots and shoots, whereas the minimum phenolic content was observed at the lowest concentration, i.e., 25.4% and 1.69%, respectively. However, in fungicide treatment, the activity increased to be 3.34- and 2.67-fold in roots and shoots, respectively.

Quantitative changes in plant defense were also observed for PAL activity in roots and shoots treated with various concentrations of MLE-AgNPs (Figure 14B). In case of PAL activity, maximum amounts were observed in plants treated with higher concentrations. There was an increase of 1.91- and 2.09-fold in roots and shoots at 120 μg/ml as compared to the pathogen control. Similarly, an increase of 1.76- and 1.83-fold was observed for roots and shoots in the non-pathogen control, whereas the lowest quantity of PAL activity was detected in the pathogen control. A wave-like pattern in terms of increasing and decreasing trends was observed for PAL activity.

The highest PO activity was exhibited at higher concentrations of MLE-AgNPs in roots and shoots (Figure 14C). The highest activity was observed at 100 and 120 μg/ml, with an increase of 1.28- and 1.27-fold in roots and 1.30- and 1.28-fold in shoots as compared to the control. Other concentration ranges from 10 to 80 μg/ml both in roots and shoots exhibited 5.5–25.3% and 10–26.1%, respectively. Non-pathogen control also showed an increase in PO activity by 1.23-fold in roots and 1.22-fold in shoots as compared to the pathogen control; however, at the highest concentrations, i.e., 140 μg/ml, a comparatively reduced activity was noted. The increase in PO activity of the treated tomato plants followed the same trend as phenolic activity. In case of PPO activity, excessive amounts were observed in tomato plants (roots and shoots) treated with various concentrations of MLE-AgNPs (Figure 14D). Various concentration ranges from 5 to 140 μg/ml exhibited 8.8–99.6% activity in roots and 1.49–84.1% in shoots, while maximum activity was observed at 120 μg/ml as there was an increase of 2.00-fold in roots and 1.84-fold in shoots as compared to the control. Lower concentrations, i.e., 40–80 μg/ml, also showed higher PPO activity in comparison to the control and non-pathogen control. These results showed that AgNPs could induce a higher amount of PPO activity as compared to the fungicide treated.

## Discussion

The green synthesis of NPs has currently established an environment-friendly and safest method involving a wide range of biological entities including fungi, bacteria, and different plant parts. UV-vis spectroscopy is an important tool to inspect the formation and steadiness of metal NPs as absorption band is detected in UV-vis range due to the SPR of metal electrons in NP solution which ultimately gives information regarding the shape and size of the NPs (Link and El-Sayed, 2003; Noginov et al., 2006). The change in color regulates the synthesis of AgNPs from the aqueous extract of *M. azedarach* (Elumalai et al., 2010). The reduction of silver metal ions A^+^ into AgNPs is due to the presence of active molecules in the leaf extract (Ahmad et al., 2003). Most probably, the speedy rate of reaction by using microwave irradiations is due to constant and rapid heating of the medium, which gives homogeneous nucleation and synthesis of NPs (Ali K. et al., 2015). Integration of microwave chemistry with biosynthetic methods significantly enhances the synthesis of NPs without interrupting green reaction conditions (Joseph and Mathew, 2015). Our results support the observation of Francis et al. (2018), who recently reported on the formation of stable AgNPs from the leaf extract of *Elephantopus scaber* by using domestic oven irradiation. Active biomolecules present in the aqueous extract of *M. azedarach* interceded the formation of AgNPs (Tripathy et al., 2010; Sukirtha et al., 2011). There are evidences which state that gallic acid results in rapid reduction of silver nitrate into AgNPs which is due to hydrolysis of tannic acid into gallic acid and glucose under a slightly acidic and basic environment (Martinez-Castanon et al., 2008). Our results are in accordance to Ali K. et al. (2015) who synthesized AgNPs after 30 s of microwave irradiation. The UV-vis spectral analysis predicts that NPs are evenly distributed and somehow more spherical in shape. Small, uniform-sized NPs can be yielded by microwave synthesis in relatively lesser time, and that is the main attraction of this method. The rapid utilization of the initial materials minimizes the chances of agglomeration during microwave-assisted methods and results in narrow-sized distributed NPs (Nadagouda et al., 2011). Increasing pH of the reaction mixture results in an increased reduction of silver nitrate (AgNO_3_) by aqueous MLE. This may be attributed to the fact that at higher pH, more H^+^ ions are available, which results in faster reduction of AgNO_3_ and oxidation of the metabolites (Jayapriya and Lalitha, 2013). Parameters such as temperature and pH can be used to modify the size and shape of AgNPs, exclusive of any additional stabilizers, as cellular proteins themselves act as stabilizing agents (Aziz et al., 2019). Disparity in the metal salt concentrations and biological material influences the NP synthesis (Pimprikar et al., 2009). It is therefore suggested that microwave-assisted green synthesis by using MLEs acts as both capping and reducing agent and has been subjected for one pot green synthesis of MLE-AgNPs at rapid rate due to improved product yield, consuming less energy, reducing reaction time, comparatively smaller size, as it has been reported previously (Nadagouda et al., 2011; Ali K. et al., 2015). FTIR confirms the absorption of characteristic frequencies of IR radiations of different functional groups (Kalainila et al., 2014). The results of the current investigation are in accordance to previous studies (Raghunandan et al., 2011; Ali K. et al., 2015) that suggested that the peak from 1,607 to 1,636 cm^–1^ is due to C=C aromatic ring stretching vibrations. However, Satyavani et al. (2011) proposed that the peaks at 1,620–1,636 cm^–1^ represent carbonyl group (C=O) present in polyphenols such as theaflavin, epicatechin gallate, and catechin gallate. The major sources for these vibration bands are various phytochemicals, flavonoids, and alkaloids that are copiously present in the leaf. Biogenic AgNPs are capped by amine and amide group; in addition to, extracts are responsible for reduction of Ag^+^ to Ag^0^, involving the formation of transitional complexes with phenolic OH groups, FTIR insinuates that the presence of peptides on the surface of biogenic AgNPs plays a pivotal role in the synthesis and capping of NPs (Aziz et al., 2015). Few unassigned peaks in XRD pattern indicate the presence of some bio-organic proteins/compounds in the leaf extracts that crystallize on the surface of the AgNPs (Jemal et al., 2017). Ramanibai and Velayutham (2015) performed XRD for AgNPs synthesized from *M. azedarach*, and the calculated particle size was 22 nm, which is very close to our study. Size variability depends on many factors such as pH, temperature, and type of plant extracts (Musarrat et al., 2010). Previous reports showed various size ranges (0.5–350 nm) for AgNPs synthesized from different leaf extracts (Ahmed et al., 2016). Generally, it is common that due to SPR, silver nano-crystals showed a typical absorption peak at 3 keV in EDX (Magudapatty et al., 2001). Some other minor peaks in graph may be possible due to the presence of biomolecules in plant material bound to AgNPs (Reddy et al., 2015). The SEM results of the current study are in accordance with the work of Mehmood et al. (2017), who synthesized predominantly spherical shaped AgNPs from *M. azedarach* extract. The various sizes of the particles may be related with different shapes and may be due to aggregation of the smaller ones.

Dynamic light scattering is used to investigate polydispersity or monodispersity and quantitative size distribution of NPs in the colloidal solution. Sample preparations are mainly involved in variation of results (Zia et al., 2016). DLS results of the present study revealed larger particle size and polydispersity in comparison to TEM analysis which mainly included the size of capping agent that lids the surface of AgNPs (Bhakya et al., 2016). Zeta potential is an important parameter indicating the long-term stability and state of NP in dispersion. In accordance to previous literature, highly stable NPs have zeta potential values that range from greater than +25 mV or less than −25 mV. Due to aggregation of interparticle attractions, dispersions showed low zeta potential values; however, it also depends on solution pH and electrolyte concentration (Sukirtha et al., 2012). The zeta potential (−22.3 mV) for MLE-AgNPs in the current study indicates high stability which could be attributed to the presence of organic coating formed around the NPs.

Transmission electron microscope images also depict the AgNP distribution (Nagati et al., 2012). Coating with capping agent signifying the presence of biomolecules in the leaf extract like flavonoids, proteins, and polyphenols indicates the stabilization of NPs and prevents agglomeration even within aggregates (Pugazhendhi et al., 2015). This type of inherent capping agents allows perfect dispersion of NPs in bio reduced colloidal solution (Kathiresan et al., 2010).

The present study specifies that various concentrations of AgNPs from lower to higher significantly inhibited the radial growth of *F. oxysporum* and found to be more efficient which are in accordance with previous studies (e.g., Wani et al., 2012). Different studies reported antifungal activities of the AgNPs (Vivek et al., 2011). Various researchers worked on the synthesis of NPs from different plant extracts to explore their antifungal potential. Phull et al. (2016) studied antifungal activity of AgNPs synthesized from *Bergenia ciliata* against various fungi, and their results indicated that the NPs were more effective as compared to the *B. ciliata* extract alone. The bio-fabricated AgNPs at different concentrations accounted for complete inhibition of conidial germination of *Bipolaris sorokiniana*, spot blotch pathogen of wheat, whereas 100% conidial germination was observed in the absence of these NPs (Mishra et al., 2014). Balashanmugam et al. (2016) attempted to synthesize efficient and stable biocompatible AgNPs from the aqueous leaf extract of *Cassia roxburghii* which exhibited higher antifungal activity against plant pathogenic fungi including *F. oxysporum, Curvularia* sp., and *Rhizoctonia solani* as compared to the conventional antifungal drug, so their findings suggest that these phyto-synthesized NPs have potential to be used as an effective growth inhibitor. AgNPs exhibited antifungal activity by inactivating the sulfhydryl groups in the fungal cell wall by forming insoluble compounds, while disruption of membrane-bound lipids and enzymes resulted in lysis of the cell (Duran et al., 2005). AgNPs expressed antifungal activity by destructing the membrane integrity of the pathogen (Elgorban et al., 2016). In a former study, Krishnaraj et al. (2012) stated that AgNPs exhibited better antifungal activity against various phytopathogenic fungi. Some other authors also reported that the antimicrobial process may involve the creation of pores by binding AgNPs to the external proteins, intrusive with replication of DNA or forming ROS (Duran et al., 2016; Ottoni et al., 2017). Biogenic nanoparticles coupled with their intrinsic properties are suitable for antimicrobial activities. The synergistic effect of green synthesized AgNPs combining with fungicides was explored against three pathologically reported fungal strains, and this showed significant resistance against infectious agents (Aziz et al., 2016). Elamawi et al. (2018) evaluated the antifungal activity by estimating 90% reduction of colony-forming phytopathogenic fungi including *Fusarium moniliforme*, *F. verticillioides, Penicillium brevicompactum, Pyricularia oryzae*, and *Helminthosporium oryzae* by using AgNPs.

The SEM is extensively used to illustrate the direct interactions between NPs and biological materials (Chen et al., 2014). Disruption of fungal hyphae and cell wall of *F. oxysporum* at different concentrations of MLE-AgNPs induced toxic effects on fungal cell wall which can be attributed to the disorder in the biosynthesis of chitin. The fungal cell wall has complex dynamics and primarily composed of chitin, glycoproteins, mannans, and glucans. Disturbance of chitin synthesis results in outcomes in the form of wall disintegration and deformed and osmotically unstable fungal cells (Bowman and Free, 2006). It has been insinuated that silver ions are emancipated to the growth media from MLE-AgNPs, which attach on the surface of the cell wall by diffusing into fungal cells (Pariona et al., 2019). SEM monographs in the current study clearly show the deformation of mycelium by the action of nanomaterials; it clearly indicates morphological changes in cell such as surface damage and inflammation as well as loss of cell wall integrity. Similarly, Ouda (2014) studied some fungi and observed a detrimental effect of silver and copper NPs on fungal hyphae. Oxidative stress induced by AgNPs on fungal cells was also suggested as one of the key mechanisms for enhancing the cytotoxic effect. The formation and stable dispersion of the NPs affect their antimicrobial activity; however, during the interaction of biological cells and NPs, excretion of silver and increased residence time enhance the activity (Stark, 2011; Li et al., 2013). Eventually, the NPs inhibited the spore germination, leading to hyphal mutilation and impeding sporulation.

Propidium iodide staining of the mycelium indicated the extent of cell death and suggested that MLE-AgNPs have the ability for causing irretrievable damage and disintegration of cellular membranes by altering the membrane permeability. SEM observations are confirmed by these findings. Fungicidal effect of AgNPs against *F. oxysporum* was evidenced by expressing a high level of red fluorescence of PI intensity that was directly proportional to the exposed dose of MLE-AgNPs. As an example, the mycelium of *F. oxysporum* showed cell death and loss of membrane integrity after uptake of PI upon treatment with lawsone (Dananjaya et al., 2017). Similarly, DCFH-DA fluorescence specifies intracellular ROS production in fungal mycelium. ROS is naturally available in the cells as highly reactive signaling molecules leading to cell death by inducing oxidative stress responses in fungi and bacteria and thus serves as an indicator for depicting the physiological status of the cell (Dupre-Crochet et al., 2013; Dayem et al., 2017). These ROS are a set of ephemeral reactive-oxidants, encompassing hydrogen peroxide (H_2_O_2_), hydroxyl-radical (–OH), singlet oxygen, superoxide radical, etc. (Liu et al., 2011). Chen et al. (2016) proposed that ROS induces inhibition and morphological damage to fungal spores when GO-AgNPs comes in contact with fungal mycelium, which also confirms current observations.

Elmer and White (2016) proposed that NPs have a great potential to play an important role in agriculture sector while their studies on the effect of different types of NPs on tomatoes and eggplants both under greenhouse and field conditions suggested that plant weight and yield are improved even when they are grown in disease-infested soils. The effects of AgNPs on disease reduction and growth parameters are more pronounced at higher concentrations. Similarly, Kim et al. (2012) stated that fungal inhibition amplifies with increased concentration of AgNPs. This phenomenon arises due to higher density of AgNPs solution which has a property to saturate, adhere to the fungal hyphae, and disengage the pathogen invasion. Elmer and White (2016) studied the effect of foliar treated micronutrient NPs in disease-infested soil under greenhouse and field on tomatoes and eggplants and found that NPs performed well and affected plant yield and weight positively. Micronutrients have reduced mobility in plants especially in neutral soils, yet they play a key role in root health, so NPs have potential of micronutrients to sustain roots in disease-infected soils, but it also depends on application efficacy; therefore, NPs have a great potential to be a part of agriculture sector (Elmer and White, 2016). Madbouly et al. (2017) investigated *in vivo* and *in vitro* effects of biosynthesized AgNPs against *Fusarium* wilt, and their findings suggested that these NPs have antifungal potency to be used as synthetic fungicides as they enhanced growth parameters especially the roots when treated with various concentrations and reduced disease severity by 90% by minimizing the number of wilted seedlings. It was also proposed that coating of seedlings with AgNPs may provoke plant growth and prevent the intrusion of phytopathogens (Abdelmalek and Salaheldin, 2016). The antifungal potency of AgNPs provides a shielding effect around the roots and acts as a barrier to invasive fungi by preventing colonization and development of wilt symptoms. Incubation time also affected the treatment of seedlings with AgNPs, as longer times allowed complete saturation of seedling roots and hence play a key role in controlling the wilt disease (Madbouly, 2018).

Pandey et al. (2014) observed that with increasing concentrations of AgNPs, the chlorophyll content also increased. The findings of the present work are in accordance to these results. Farghaly and Nafady (2015) noticed significant stimulation for Chl “b” and carotenoids in tomato plants for biosynthesized AgNPs while they noted stress effect by measuring reduced Chl “a” after 35 days of treatments. Similarly, Pak et al. (2017) proposed that carotenoid content increased in *Dracocephalum moldavica* with increasing concentrations of AgNPs that protect the plant against ROS. Thus, antioxidant properties of carotenoids provide a protective function against free radicals of metallic and metal NPs (Sakihama and Yamasaki, 2002). Elbeshehy et al. (2014) studied that systemic resistance was induced in *Vicia faba* against bean yellow mosaic virus (BYMV) by using biosynthesized AgNPs and results in increased concentration of photosynthetic pigments, whereas it was decreased in infected plants likewise. Postinfection treatment of AgNPs resulted in reduced percentage infection, disease severity, and virus concentration.

Different functions of plants such as defensive approach from biocidal and herbivory against fungal and bacterial pathogens as well as structural stability are related to phenolic activity (Heldt, 1997). Virus-infected broad bean leaves treated with AgNPs indicated maximum accumulation of phenolic contents in comparison to the infected leaves at different levels of treatments (Elbeshehy et al., 2014). From our study, it was clear that disease severity was reduced with increased activities of phenolics in tomato plants treated with different concentrations of AgNPs, so the infected roots of treated tomato seedlings were found to be more resistant as compared to the control ones.

Therefore, a noteworthy increase in the activities of phenolics, PO, PPO, and PAL at the higher concentration of MLE-AgNPs is usually regarded as tolerance indicator in tomato plants. So, these results suggest that biosynthesized AgNPs enhance the level of antioxidant enzymes which may act like a defensive mechanism against oxidative stress due to disease, whereas healthy growth of plant was associated with antioxidant defenses. Like current observations, Kumari et al. (2017) found that biosynthesized AgNPs are able to diminish the pathogenic population of *Alternaria solani*, the causative agent of early blight in tomato in a concentration-dependent manner and reported that pretreatment of particles on leaves increased the host resistance and prevents the infection by increasing the antioxidant and chlorophyll content. Plant resistance was enhanced by improved enzyme activities involved in phenyl-propenoid pathway viz: PAL, PO, and POO (Trotel-Aziz et al., 2008; Jourdan et al., 2009; Radjacommare et al., 2010; Akram and Anjum, 2011). The increased activity of PO, PPO, and PAL might be associated with cell wall strengthening and production of certain bioactive compounds including lignin, suberin, quinones, and melanin which act like a protective shield for the approaching pathogens by destroying their pectolytic enzymes (Kuzniak and Urbanek, 2000; Gomez-Vasquez et al., 2004; Fugate et al., 2016), while PPO plays a key role in initiation of defense resistance against plant diseases by catalyzing phenolic oxidation (Thipyapong and Stiffens, 1997). Pak et al. (2017) proposed that silver accumulated in root tissues after AgNP treatment mainly exists in the form of NPs which were highly stable and did not release ionic silver after inflowing the cells, so they showed least toxicity in comparison to ionic silver.

## Conclusion

Nanotechnology plays a dynamic role in introducing multiple approaches for suppressing disease, enhancing disease diagnostics, and developing new measures for manipulation of plants and pathogens. Thus, biosynthesized MLE-AgNPs is an eco-friendly approach to control *Fusarium* wilt of tomatoes at various concentrations by suppressing the growth of *F. oxysporum*. MLE-AgNPs has shown strong potential to restrain the fungal population both in lab and field trials in dose-dependent manner. Application of NPs on pre-infected roots of tomato plants successfully reduced the wilt by increasing the resistance of the host plant and enhancing the growth parameters of tomato seedlings. Photosynthetic pigments and antioxidant enzymes increased with the various concentrations of MLE-AgNPs. So, this study provides a basis that biosynthesized NPs can be used as an alternative to conventional fungicides and become helpful in minimizing environmental pollution. Hence, they have the ability to replace the health hazards of chemical fungicides.

## Data Availability Statement

The datasets generated for this study are available on request to the corresponding author.

## Author Contributions

TA and HA designed the study. HA has worked as a research scholar for this project. SR and SN have provided technical guidance for various analyses. All the authors read and approved the final version of the manuscript.

## Conflict of Interest

The authors declare that the research was conducted in the absence of any commercial or financial relationships that could be construed as a potential conflict of interest.

## References

[B1] Abdelmalek G. A. M. Salaheldin T. A. (2016). Silver nanoparticles as a potent fungicide for citrus phytopathogenic fungi. *J. Nanomed. Res.* 3:00065 10.15406/jnmr.2016.03.00065

[B2] Aguilar-Mendez M. A. Martin-Martinez E. S. Ortega-Arroyo L. Cobian-Portillo G. Sanchez-Espindola E. (2011). Synthesis and characterization of silver nanoparticles: effect on phytopathogen *Colletotrichum gloesporioides*. *J. Nanopart. Res.* 13 2525–2532. 10.1007/s11051-010-0145-6

[B3] Ahmad A. Mukherjee P. Senapati S. Mandal D. Khan M. I. Kumar R. (2003). Extracellular biosynthesis of silver nanoparticles using the fungus *Fusarium oxysporum*. *Collodis Surf. B* 28 313–318. 10.1016/S0927-7765(02)00174-1

[B4] Ahmed S. Saifullah, Ahmad M. Swami B. L. Ikram S. (2016). Green synthesis of silver nanoparticles using *Azadirachta indica* aqueous leaf extract. *J. Radiat. Res. Appl. Sci.* 9 1–7. 10.1016/j.jrras.2015.06.006

[B5] Akram W. Anjum T. (2011). Use of bioagents and synthetic chemicals for induction of systemic resistance in tomato against diseases. *Int. R. J. Agric. Sci. Soil Sci.* 1 286–292.

[B6] Ali K. Ahmed B. Dwivedi S. Saquib Q. AlKhedhairy A. A. Musarrat J. (2015). Microwave accelerated green synthesis of stable silver nanoparticles with *Eucalyptus globulus* leaf extract and their antibacterial and antibiofilm activity on clinical isolates. *PLoS One* 10:e0131178. 10.1371/journal.pone.0131178 26132199 PMC4489395

[B7] Ali M. Kim S. Belfield K. Norman D. Brennan M. Ali G. (2015). Inhibition of *Phytophthora* spp. by silver nanoparticles synthesized using aqueous extract of *Artemisia absinthium*. *Phytopathology* 105 1183–1190. 10.1094/phyto-01-15-0006-r 25871856

[B8] Austin L. A. Mackey M. A. Dreaden E. C. El-Sayed M. A. (2014). The optical, photothermal, and facile surface chemical properties of gold and silver nanoparticles in biodiagnostics, therapy, and drug delivery. *Arch. Toxicol.* 88 1391–1417. 10.1007/s00204-014-1245-3 24894431 PMC4136654

[B9] Aziz N. Faraz M. Pandey R. Shakir M. Fatma T. Varma A. (2015). Facile algae-derived route to biogenic silver nanoparticles: synthesis, antibacterial, and photocatalytic properties. *Langmuir* 31 11605–11612. 10.1021/acs.langmuir.5b03081 26447769

[B10] Aziz N. Faraz M. Sherwani M. A. Fatma T. Prasad R. (2019). Illuminating the anticancerous efficacy of a new fungal chassis for silver nanoparticle synthesis. *Front. Chem.* 7:65. 10.3389/fchem.2019.00065 30800654 PMC6375905

[B11] Aziz N. Pandey R. Barman I. Prasad R. (2016). Leveraging the attributes of *Mucor hiemalis*-derived silver nanoparticles for a synergistic broad-spectrum antimicrobial platform. *Front. Microbiol.* 7:1984. 10.3389/fmicb.2016.01984 28018316 PMC5156874

[B12] Balashanmugam P. Balakumaran M. D. Murugan R. Dhanapal K. Kalaichelvan P. T. (2016). Phytogenic synthesis of silver nanoparticles, optimization and evaluation of in vitro antifungal activity against human and plant pathogens. *Microbiol. Res.* 192 52–64. 10.1016/j.micres.2016.06.004 27664723

[B13] Bhakya S. Muthukrishnan S. Sukumaran M. Muthukumar M. (2016). Biogenic synthesis of silver nanoparticles and their antioxidant and antibacterial activity. *Appl. Nanosci.* 6 755–766. 10.1007/s13204-015-0473-z

[B14] Bowman S. M. Free S. J. (2006). The structure and synthesis of the fungal cell wall. *Bioessays* 28 799–808. 10.1002/bies.20441 16927300

[B15] Burrell M. M. Rees T. A. (1974). Metabolism of phenylalanine and tyrosine in rice leaves infected by *Pyricularia oryzae*. *Physiol. Plant Pathol.* 4 497–474. 10.1016/j.micres.2016.06.004

[B16] Chen J. Peng H. Wang X. Shao F. Yuan Z. Han H. (2014). Graphene oxide exhibits broad-spectrum antimicrobial activity against bacterial phytopathogens and fungal conidia by intertwining and membrane perturbation. *Nanoscale* 6 1879–1889. 10.1039/c3nr04941h 24362636

[B17] Chen J. Sun L. Cheng Y. Lu Z. Shao K. Li T. (2016). Graphene oxide-silver nanocomposite: novel agricultural antifungal agent against *Fusarium graminearum* for crop disease prevention. *ACS Appl. Mater. Interfaces* 8 24057–24070. 10.1021/acsami.6b05730 27563750

[B18] Chung I. M. Park I. Seung-Hyun K. Thiruvengadam M. Rajakumar G. (2016). Plant-mediated synthesis of silver nanoparticles: their characteristic properties and therapeutic applications. *Nanoscale Res. Lett.* 11:40. 10.1186/s11671-016-1257-4 26821160 PMC4731379

[B19] Dananjaya S. H. S. Udayangani R. M. C. Shin S. Y. Edussuriya M. Nikapitiya C. Lee J. (2017). In vitro and in vivo antifungal efficacy of plant based lawsone against *Fusarium oxysporum* species complex. *Microbiol. Res.* 201 21–29. 10.1016/j.micres.2017.04.011 28602398

[B20] Dayem A. A. Hossain M. K. Lee S. B. Kim K. Saha S. K. Yang G. M. (2017). The role of reactive oxygen species (ROS) in the biological activities of metallic nanoparticles. *Int. J. Mol. Sci.* 18:E120 10.3390/ijms18010120 PMC5297754 28075405

[B21] Duan H. Wang D. Li Y. (2015). Green chemistry for nanoparticle synthesis. *Chem. Soc. Rev.* 44 5778–5792. 10.1007/s00449-014-1251-0 25615873

[B22] Dupre-Crochet S. Erard M. Nüβe O. (2013). ROS production in phagocytes: why, when, and where? *J. Leukoc. Biol* 94 657–670. 10.1189/jlb.1012544 23610146

[B23] Duran N. Marcato P. D. Alves O. L. Souza G. Esposito E. (2005). Mechanistic aspects of biosynthesis of silver nanoparticles by several *Fusarium oxysporum* strains. *J. Nanobiotechnol.* 3 1–8. 10.1186/1477-3155-3-8 16014167 PMC1180851

[B24] Duran N. Nakazato G. Seabra A. B. (2016). Antimicrobial activity of biogenic silver nanoparticles, and silver chloride nanoparticles: an overview and comments. *Appl. Microbiol. Biotechnol.* 100 6555–6570. 10.1007/s00253-016-7657-7 27289481

[B25] Elamawi R. M. Al-Harbi R. E. Hendi A. A. (2018). Biosynthesis and characterization of silver nanoparticles using *Trichoderma longibrachiatum* and their effect on phytopathogenic fungi. *Egypt. J. Biol. Pest Control* 28:28 10.1186/s41938-018-0028-1

[B26] Elbeshehy E. K. F. Almaghrabi O. A. Mahmoud W. M. A. Elazzazy A. M. (2014). Effect of biosynthesized silver nanoparticles on physiological parameters of *Vicia faba* infected by bean yellow mosaic virus. *J. Pure Appl. Microbiol.* 8 803–812.

[B27] Elgorban A. M. El-Samawaty A. M. Yassin M. A. Sayed S. R. Adil S. F. Elhindi K. M. (2016). Antifungal silver nanoparticles: synthesis, characterization and biological evaluation. *Biotechnol. Biotechnol. Equip.* 30 56–62. 10.1080/13102818.2015.1106339

[B28] Elmer W. H. White J. C. (2016). The use of metallic oxide nanoparticles to enhance growth of tomatoes and eggplants in disease infested soil or soilless medium. *Environ. Sci. Nano* 3 1072–1079. 10.1039/C6EN00146G

[B29] El-Mohamedy R. S. D. Jabnoun-Khiareddine H. Daami-Remadi M. (2014). Control of root rot diseases of tomato plants caused by *Fusarium solani*, *Rhizoctonia solani* and *Sclerotium rolfsii*. *Tunisian J. Plant. Prot.* 9 45–55.

[B30] El-Mougy N. S. Abdel-Kader M. M. Lashin S. M. Megahed A. A. (2013). Fungicides alternatives as plant resistance inducers against foliar diseases incidence of some vegetables grown under plastic houses conditions. *Int. J. Eng. Innovat. Technol.* 3 71–81.

[B31] El-Temsah Y. S. Oughton D. H. Joner E. J. (2014). Effects of nano-sized zero-valent iron on DDT degradation and residual toxicity in soil: a column experiment. *Plant Soil* 368 189–200. 10.1016/j.chemosphere

[B32] Elumalai E. K. Prasad T. N. V. K. V. Hemachandran J. Viviyan Therasa S. Thirumalai T. David E. (2010). Extracellular synthesis of silver nanoparticles using leaves of *Euphorbia hirta* and their antibacterial activities. *J. Pharm. Sci. Res.* 2 549–554.

[B33] FAOSTAT (2014). *Global Tomato Production in 2012.* Rome: FAO.

[B34] Farghaly A. Nafady N. A. (2015). Green Synthesis of Silver Nanoparticles using leaf extract of *Rosmarinus officinalis* and its effect on tomato and wheat plants. *J. Agric. Sci.* 7 277–287. 10.5539/jas.v7n11p277

[B35] Francis S. Joseph S. Ebey P. Koshy E. P. Mathew B. (2018). Microwave assisted green synthesis of silver nanoparticles using leaf extract of *elephantopus scaber* and its environmental and biological applications. *Artif. Cells Nanomed. Biotechnol.* 46 795–804. 10.1080/21691401.2017.1345921 28681662

[B36] Fu J. M. Huang B. R. (2001). Involvement of antioxidants and lipid peroxidation in the adaptation of two cool-season grasses to localized drought stress. *Environ. Exp. Bot.* 45 105–114. 10.1016/s0098-8472(00)00084-8 11275219

[B37] Fugate K. K. Ribeiro W. S. Lulai E. C. Deckard E. L. Finger F. L. (2016). Cold temperature delays wound healing in postharvest sugarbeet roots. *Front. Plant Sci.* 7:499. 10.3389/fpls.2016.00499 27148322 PMC4830815

[B38] Ghrair A. M. Ingwersen J. Streck T. (2010). Immobilization of heavy metals in soils amended by nanoparticulate zeolitic tuff: sorption-desorption of cadmium. *J. Plant Nutr. Soil Sci.* 173 852–860. 10.1002/jpln.200900053

[B39] Gomez-Vasquez R. Day R. Buschmann H. Randles S. Beeching J. R. Cooper R. M. (2004). Phenylpropanoids, phenylalanine ammonia lyase and peroxidases in elicitor-challenged cassava (Manihot esculenta) suspension cells and leaves. *Ann. Bot.* 94 87–97. 10.1093/aob/mch107 15145789 PMC4242363

[B40] GOP (2013). *Fruit, Vegetables and Condiments Statistics of Pakistan 2011-12.* Islamabad: Government of Pakistan Ministry of National Food Security and Research.

[B41] Heldt H. W. (1997). *Plant Biochemistry and Molecular Biology*, 1st Edn Oxford: Oxford University Press.

[B42] Jamshidi M. Ghanati F. (2016). Taxanes content and cytotoxicity of hazel cells extract after elicitation with silver nanoparticles. *Plant Physiol. Biochem.* 110 178–184. 10.1016/j.plaphy.2016.04.026 27112786

[B43] Jasim B. Thomas R. Mathew J. Radhakrishnan E. K. (2017). Plant growth and diosgenin enhancement effect of silver nanoparticles in Fenugreek (*Trigonella foenum-graecum* L.). *Saudi Pharm. J.* 25 443–447. 10.1016/j.jsps.2016.09.012 28344500 PMC5357095

[B44] Jayapriya E. Lalitha P. (2013). Synthesis of silver nanoparticles using leaf aqueous extract of *Ocimum basilicum* (L.). *Int. J. Chem Tech Res.* 5 2985–2292.

[B45] Jemal K. Sandeep B. V. Pola S. (2017). Synthesis, characterization, and evaluation of the antibacterial activity of *Allophylus serratus* leaf and leaf derived callus extracts mediated silver nanoparticles. *J. Nanomater.* 2017 1–11. 10.1155/2017/4213275

[B46] Jensen C. R. Battilani A. Plauborg F. Psarras G. Chartzoulakis K. Janowiak F. (2010). Deficit irrigation based on drought tolerance and root signalling in potatoes and tomatoes. *Agric. Water Manag.* 98 403–413. 10.1016/j.agwat.2010.10.018

[B47] Joseph S. Mathew B. (2015). Microwave-assisted green synthesis of silver nanoparticles and the study on catalytic activity in the degradation of dyes. *J. Mol. Liq.* 204 184–191. 10.1016/j.molliq.2015.01.027 30104462

[B48] Jourdan E. Henry G. Duby F. Dommes J. Barthelemy J. P. Thonart P. (2009). Insights into the defense-related events occurring in plant cells following perception of surfactin-type lipopeptide from *Bacillus subtilis*. *Mol. Plant Microbe Interact.* 22 456–468. 10.1094/MPMI-22-4-0456 19271960

[B49] Jung J. H. Kim S. W. Min J. S. Kim Y. J. Lamsal K. Kim K. S. (2010). The effect of nano-silver liquid against the white rot of the green onion caused by *Sclerotium cepivorum*. *Mycobiology* 38 39–45. 10.4489/MYCO.2010.38.1.039 23956623 PMC3741593

[B50] Kalainila P. Subha V. Ernest Ravindran R. S. Sahadevan R. (2014). Synthesis and characterization of silver nanoparticles from *Erythrina indica*. *Asian J. Pharm. Clin. Res.* 7 39–43. 10.1166/jnn.2012.5926 25189525

[B51] Kathiresan K. Alikunhi N. M. Pathmanaban S. Nabikhan A. Kandasamy S. (2010). Analysis of antimicrobial silver nanoparticles synthesized by coastal strains of *Escherichia coli* and *Aspergillus niger*. *Can. J. Microbiol.* 1056 1050–1059. 10.1139/W10-094 21164575

[B52] Khot L. R. Sankaran S. Maja J. M. Ehsani R. Schuster E. W. (2012). Applications of nanomaterials in agricultural production and crop protection: a review. *Crop Prot.* 35 64–70. 10.1016/j.cropro.2012.01.007

[B53] Kim S. W. Jung J. H. Lamsal K. Kim Y. S. Min J. S. Lee Y. S. (2012). Antifungal effects of silver nanoparticles (AgNPs) against various plant pathogenic fungi. *Mycobiology* 40 53–58. 10.5941/myco.2012.40.1.053 22783135 PMC3385153

[B54] Krishnaraj C. Ramachandran R. Mohan K. Kalaichelvan P. T. (2012). Optimization for rapid synthesis of silver nanoparticles and its effect on phytopathogenic fungi. *Spectrochim. Acta* 93 95–99. 10.1016/j.saa.2012.03.002 22465774

[B55] Kumari M. Pandey S. Bhattacharya A. Mishra A. Nautiyal C. S. (2017). Protective role of biosynthesized silver nanoparticles against early blight disease in *Solanum lycopersicum*. *Plant Physiol. Biochem.* 121 216–225. 10.1016/j.plaphy.2017.11.004 29149700

[B56] Kuzniak E. Urbanek H. (2000). The involvement of hydrogen peroxide in plant responses to stresses. *Acta Physiol. Plant.* 22 195–203. 10.1007/s11738-000-0076-4

[B57] Li C. Wang X. Chen F. Zhang C. Zhi X. Wang K. (2013). The antifungal activity of graphene oxide-silver nanocomposites. *Biomaterials* 34 3882–3890. 10.1016/j.biomaterials.2013.02.001 23465487

[B58] Link S. El-Sayed M. A. (2003). Optical properties and ultrafast dynamics of metallic nanocrystals. *Annu. Rev. Phys. Chem.* 54 331–366. 10.1146/annurev.physchem.54.011002.103759 12626731

[B59] Liu S. Zeng T. H. Hofmann M. Burcombe E. Wei J. Jiang R. (2011). Antibacterial activity of graphite, graphite oxide, graphene oxide, and reduced graphene oxide: membrane and oxidative stress. *ACS Nano* 5 6971–6980. 10.1021/nn202451x 21851105

[B60] Madbouly A. K. (2018). Nanoparticles as novel plant growth promoters. *Novel Res. Microbiol. J.* 2, 61–64. 10.21608/NRMJ.2018.12547

[B61] Madbouly A. K. Mohamed S. Abdel-Aziz M. S. Abdel-Wahhab M. A. (2017). Biosynthesis of nanosilver using *Chaetomium globosum* and its application to control Fusarium wilt of tomato in the greenhouse. *IET Nanobiotechnol.* 11 702–708. 10.1049/iet-nbt.2016.0213

[B62] Magudapatty P. Gangopadhgayrans P. Panigrahi B. K. Nair K. G. M. Dhara S. (2001). Electrical transport studies of Ag nanoclusters embedded in glass matrix. *Physica B Condens. Matter* 299, 142–146. 10.1016/S0921-4526(00)00580-9

[B63] Majdalawieh A. Kanan M. C. El-Kadri O. Kanan S. M. (2014). Recent advances in gold and silver nanoparticles: synthesis and applications. *J. Nanosci. Nanotechnol.* 14 4757–4780. 10.1166/jnn.2014.9526 24757945

[B64] Mallaiah B. (2015). *Integrate Approaches for the Management of Crossandra (Crossandra infundibuliformis L. nees) Wilt Caused by Fusarium in carnatum (Desm.) Sacc.* Ph.D. thesis, Tamil Nadu Agriculture University, Madurai.

[B65] Martinez-Castanon G. A. Nino-Martinez N. Martinez-Gutierrez F. Martinez Mendoza J. R. Ruiz F. (2008). Synthesis and antibacterial activity of silver nanoparticles with different sizes. *J. Nanopart. Res.* 10 1343–1348.

[B66] Mayer A. M. Harel E. Shaul R. B. (1966). Assay of catechol oxidase a critical comparison of methods. *Phytochemistry* 5 783–789. 10.1016/S0031-9422(00)83660-2 9877098

[B67] Medda S. Hajra A. Dey U. Bose P. Mondal N. (2015). Biosynthesis of silver nanoparticles from Aloe vera leaf extract and antifungal activity against *Rhizopus* sp. and *Aspergillus* sp. *Appl. Nanosci.* 5 875–880. 10.1007/s13204-014-0387-1

[B68] Mehmood A. Murtaza G. Bhatti T. M. Kausar R. (2017). Phyto-mediated synthesis of silver nanoparticles from *Melia azedarach* L. leaf extract: characterization and antibacterial activity. *Arab. J. Chem.* 10 3048–3053. 10.1016/j.arabjc.2013.11.046

[B69] Mishra S. Singh B. R. Singh A. Keswani C. Naqvi Singh H. B. (2014). Biofabricated silver nanoparticles act as a strong fungicide against *Bipolaris sorokiniana* causing spot blotch disease in wheat. *PLoS One* 9:e97881. 10.1371/journal.pone.0097881 24840186 PMC4026416

[B70] Mohanta Y. K. Panda S. K. Bastia A. K. Mohanta T. K. (2017). Biosynthesis of silver nanoparticles from *Protium serratum* and investigation of their potential impacts on food safety and control. *Front. Microbiol.* 8:626. 10.3389/fmicb.2017.00626 28458659 PMC5394122

[B71] Musarrat J. Dwivedi S. Singh B. R. Al-Khedhairy A. A. Azam A. Naqvi A. (2010). Production of antimicrobial silver nanoparticles in water extracts of the fungus *Amylomyces rouxii* strain KSU-09. *Bioresour. Technol.* 101 8772–8776. 10.1016/j.biortech.2010.06.065PMID:20619641 20619641

[B72] Nadagouda M. N. Speth T. F. Varma R. S. (2011). Microwave-assisted green synthesis of silver nanostructures. *Acc. Chem. Res.* 44 469–478. 10.1021/ar1001457 21526846

[B73] Nagati V. B. Rama K. Manisha R. D. Jahnavi A. Karunakar R. (2012). Green synthesis and characterization of silver nanoparticles from *Cajanus cajan* leaf extract and its antibacterial activity. *Int. J. Nanomater. Biostruct.* 2 39–43.

[B74] Noginov M. A. Zhu G. Bahoura M. Adegoke J. Small C. Ritzo B. A. (2006). The effect of gain and absorption on surface plasmon in metal nanoparticles. *Appl. Phys. B* 86 455–460. 10.1007/s00340-006-2401-0 22486413

[B75] Noruzi M. (2015). Biosynthesis of gold nanoparticles using plant extracts. *Bioprocess Biosyst. Eng.* 38 1–14. 10.1007/s00449-014-1251-0 25090979

[B76] Ottoni C. A. Simaes M. F. Fernandes S. Santos J. G. Da Silva E. S. Souza R. F. B. (2017). Screening of filamentous fungi for antimicrobial silver nanoparticles synthesis. *AMB Express* 7 31–41. 10.1186/s13568-017-0332-2 28144889 PMC5285291

[B77] Ouda S. M. (2014). Antifungal activity of silver and copper nanoparticles on two plant pathogens, *Alternaria alternata* and *Botrytis cinerea*. *Res. J. Microbiol.* 9 34–42. 10.3923/jm.2014.34.42

[B78] Oves M. Aslam M. Rauf M. A. Qayyum S. Qari H. A. Khan M. S. (2018). Antimicrobial and anticancer activities of silver nanoparticles synthesized from the root hair extract of *Phoenix dactylifera*. *Mater. Sci. Eng. C Mater. Biol. Appl.* 89 429–443. 10.1016/j.msec.2018.03.035 29752116

[B79] Pak Z. H. Karimi N. Abbaspour H. (2017). Effects of Silver nanoparticle exposure on growth, physiological and biochemical parameters of *Dracocephalum moldavica* L. *Iran. J. Plant Physiol.* 7 2173–2183. 10.22034/IJPP.2017.537982

[B80] Pandey C. Khan E. Mishra A. Sardar M. Gupta M. (2014). Silver nanoparticles and its effect on seed germination and physiology in *Brassica juncea* L. (Indian mustard) plant. *Adv. Sci. Lett.* 20 1673–1676. 10.1166/asl.2014.5518

[B81] Pariona N. Mtz-Enriquez A. I. Sanchez-Rangel D. Carrion G. Paraguay-Delgado F. Rosas-Saito G. (2019). Green-synthesized copper nanoparticles as a potential antifungal against plant pathogens. *RSC Adv.* 9 188835–118843. 10.1039/c9ra03110c PMC9065100 35516870

[B82] Phull A. R. Abbas Q. Ali A. Raza H. Kim S. J. Zia M. (2016). Antioxidant, cytotoxic and antimicrobial activities of green synthesized silver nanoparticles from crude extract of *Bergenia ciliata*. *Future J. Pharm. Sci.* 2 31–36. 10.1016/j.fjps.2016.03.001

[B83] Pimprikar P. S. Joshi S. S. Kumar A. R. Zinjarde S. S. Kulkarni S. K. (2009). Influence of biomass and gold salt concentration on nanoparticle synthesis by the tropical marine yeast *Yarrowia lipolytica* NCIM 3589. *Colloids Surf. B* 74 309–316. 10.1016/j.colsurfb.2009.07.040 19700266

[B84] Pocock T. Krol M. Huner N. P. (2004). The determination and quanti?cation of photosynthetic pigments by reverse phase high-performance liquid chromatography, thin-layer chromatography, and spectrophotometry. *Methods Mol. Biol.* 274 137–148. 10.1385/1-59259-799-8:137 15187276

[B85] Porra R. J. (2002). The chequered history of the development and use of simultaneous equations for the accurate determination of chlorophylls a and b. *Photosynth. Res.* 73 149–156. 16245116 10.1023/A:1020470224740

[B86] Prasad R. Pandey R. Barman I. (2016). Engineering tailored nanoparticles with microbes: quo vadis? *WIREs Nanomed. Nanobiotechnol.* 8 316–330. 10.1002/wnan.1363 26271947

[B87] Pugazhendhi S. Kirubha E. Palanisamy P. K. Gopalakrishnan R. (2015). Synthesis and characterization of silver nanoparticles from *Alpinia calcarata* by Green approach and its applications in bactericidal and nonlinear optics. *Appl. Surf. Sci.* 357 1801–1808. 10.1016/j.apsusc.2015.09.237

[B88] Qayyum S. Oves M. Khan A. U. (2017). Obliteration of bacterial growth and biofilm through ROS generation by facilely synthesized green silver nanoparticles. *PLoS One* 12:e0181363. 10.1371/journal.pone.0181363 28771501 PMC5542591

[B89] Radjacommare R. Venkatesan S. Samiyappan R. (2010). Biological control of phytopathogenic fungi of vanilla through lytic action of Trichoderma species and *Pseudomonas fluorescens*. *Arch. Phytopathol. Plant. Prot.* 43 1–17. 10.1080/03235400701650494

[B90] Raghunandan D. Bedre M. D. Basavaraja S. Sawle B. Manjunath S. Y. Venkataraman A. (2011). Rapid biosynthesis of irregular shaped gold nanoparticles from macerated aqueous extracellular dried clove buds (*Syzygium aromaticum*) solution. *Colloids Surf. B* 79 235–240. 10.1016/j.colsurfb.2010.04.003 20451362

[B91] Ramanibai R. Velayutham K. (2015). Bioactive compound synthesis of Ag nanoparticles from leaves of *Melia azedarach* and its control for mosquito larvae. *Res. Vet. Sci.* 98 82–88. 10.1016/j.rvsc.2014.11.009 25496834

[B92] Reddy M. C. Murthy K. S. R. Srilakshmi A. Sambasiva Rao K. R. S. Pullaiah T. (2015). Phytosynthesis of eco-friendly silver nanoparticles and biological applications–A novel concept in Nanobiotechnology. *Afr. J. Biotechnol.* 14 222–247. 10.5897/AJB2013.13299

[B93] Sakihama Y. Yamasaki H. (2002). Lipid peroxidation induced by phenolic in conjunction with aluminum ions. *Biol. Plant.* 45 249–254.

[B94] Satyavani K. Gurudeeban S. Ramanathan T. Balasubramanian T. (2011). Biomedical potential of silver nanoparticles synthesized from calli cells of *Citrullus colocynthis* (L.) schrad. *J. Nanobiotechnol.* 9 43–51. 10.1186/1477-3155-9-43 21943321 PMC3203035

[B95] Shenashen M. Derbalah A. Hamza A. Mohamed A. El-Safty S. (2017). Antifungal activity of fabricated mesoporous alumina nanoparticles against rot root disease of tomato caused by *Fusarium oxysporum*. *Pest Manag. Sci.* 73 1121–1126. 10.1002/ps.4420 27558672

[B96] Siripattanakul-Ratpukdi S. Furhacker M. (2014). Review: issues of silver nanoparticles in engineered environmental treatment systems. *Water Air Soil Pollut.* 225 1–18. 10.1007/s11270-014-1939-4

[B97] Soares M. Correa R. O. Stroppa P. Marques F. C. Andrade G. Correa C. C. (2018). Biosynthesis of silver nanoparticles using *Caesalpinia ferrea* (Tul.) Martius extract: physicochemical characterization, antifungal activity and cytotoxicity. *PeerJ* 6:e4361. 10.7717/peerj.4361 29576936 PMC5863706

[B98] Stark W. J. (2011). Nanoparticles in biological systems. *Angew. Chem. Int. Ed.* 50 1242–1258. 10.1002/anie.200906684 21290491

[B99] Sukirtha R. Krishnan M. Ramachandran R. Kamalakkannan S. Kokilavani P. SankarGanesh D. (2011). *Areca catechu* Linn. –derived silver nanoparticles: a novel antitumor agent against dalton’s ascites lymphoma. *Int. J. Green Nanotechnol.* 3 2–12. 10.1080/19430892.2011.571626

[B100] Sukirtha R. Priyanka K. M. Antony J. J. Kamalakkannan S. Thangam R. Gunasekaran P. (2012). Cytotoxic effect of Green synthesized silver nanoparticles using *Melia azedarach* against in vitro HeLa cell lines and lymphoma mice model. *Process Biochem.* 47 273–279. 10.1016/j.procbio.2011.11.003

[B101] Thipyapong P. Stiffens J. C. (1997). Tomato polyphenol oxidase differential response of the PPO F promoter to injuries and wound signals. *Plant Physiol.* 115 409–418. 10.1104/pp.115.2.409 12223816 PMC158498

[B102] Tripathy A. Raichur A. M. Chandrasekaran N. Prathna T. C. Mukherjee A. (2010). Process variables in biomimetic synthesis of silver nanoparticles by aqueous extract of *Azadirachta indica* (Neem) leaves. *J. Nanopart. Res.* 12 237–246. 10.1007/s11051-009-9602-5

[B103] Trotel-Aziz P. Couderchet M. Biagianti S. Aziz A. (2008). Characterization of new bacterial biocontrol agents Acinetobacter, Bacillus, Pantoea and *Pseudomonas* spp. mediating grapevine resistance against *Botrytis cinereal*. *Environ. Exp. Bot.* 64 21–32. 10.1016/j.envexpbot.2007.12.009

[B104] Vincent J. H. (1947). Distortion of fungal hyphae in presence of certain inhibitor. *Nature* 159:850 10.1038/159850b0 20343980

[B105] Vishwakarma K. Shweta, Upadhyay N. Singh J. Liu S. Singh V. P. (2017). Differential phytotoxic impact of plant mediated silver nanoparticles (AgNPs) and silver nitrate (AgNO_3_) on *Brassica* Sp. *Front. Plant Sci.* 8:1501. 10.3389/fpls.2017.01501 29075270 PMC5644052

[B106] Vivek M. Kumar P. S. Steffi S. Sudha S. (2011). Biogenic silver nanoparticles by *Gelidiella acerosa* extract and their antifungal effects. *Avicenna J. Med. Biotechnol*. 3 143–148. 23408653 PMC3558184

[B107] Wani A. H. Amin M. Shahnaz M. Shah M. A. (2012). Antimycotic activities of MgO, FeO and ZnO on some pathogenic fungi. *Int. J. Manuf. Mater. Mech. Eng.* 2 59–70. 10.4018/ijmmme.2012100105

[B108] Wellburn A. R. (1994). The spectral determination of chlorophylls a and b, as well as total carotenoids, using various solvents with spectrophotometers of different resolution. *J. Plant Physiol.* 144 307–313. 10.1016/S0176-1617(11)81192-2

[B109] Zia F. Ghafoor N. Iqbal M. Mehboob S. (2016). Green synthesis and characterization of silver nanoparticles using *Cydonia oblong* seed extract. *Appl. Nanosci.* 6 1023–1029. 10.1007/s13204-016-0517-z

[B110] Zieslin N. Ben-Zaken R. (1993). Peroxidase activity and presence of phenolic substances in peduncles of rose flower. *Plant Physiol. Biochem.* 31 333–339.

